# “Now I Get It!”: Eureka Experiences During the Acquisition of Mathematical Concepts

**DOI:** 10.1162/opmi_a_00116

**Published:** 2024-02-01

**Authors:** Charlotte Barot, Louise Chevalier, Lucie Martin, Véronique Izard

**Affiliations:** Université Paris Cité, INCC UMR 8002, CNRS, F-75006 Paris, France

**Keywords:** insight, concept learning, geometrical cognition, Eureka moment, Aha! moment, mathematical cognition, reflective introspection, consciousness

## Abstract

Many famous scientists have reported anecdotes where a new understanding occurred to them suddenly, in an unexpected flash. Do people generally experience such “Eureka” moments when learning science concepts? And if so, do these episodes truly vehicle sudden insights, or is this impression illusory? To address these questions, we developed a paradigm where participants were taught the mathematical concept of geodesic, which generalizes the common notion of straight line to straight trajectories drawn on curved surfaces. After studying lessons introducing this concept on the sphere, participants (N = 56) were tested on their understanding of geodesics on the sphere and on other surfaces. Our findings indicate that Eureka experiences are common when learning mathematics, with reports by 34 (61%) participants. Moreover, Eureka experiences proved an accurate description of participants’ learning, in two respects. First, Eureka experiences were associated with learning and generalization: the participants who reported experiencing Eurekas performed better at identifying counterintuitive geodesics on new surfaces. Second, and in line with the firstperson experience of a sudden insight, our findings suggest that the learning mechanisms responsible for Eureka experiences are inaccessible to reflective introspection. Specifically, reports of Eureka experiences and of participants’ confidence in their own understanding were associated with different profiles of performance, indicating that the mechanisms bringing about Eureka experiences and those informing reflective confidence were at least partially dissociated. Learning mathematical concepts thus appears to involve mechanisms that operate unconsciously, except when a key computational step is reached and a sudden insight breaks into consciousness.

## INTRODUCTION

Learning new concepts is difficult and protracted, especially in science (Brock & Taber, 2017; Carey, 2009; Chi, 2008; diSessa, 2014; Ohlsson, 2009; Özdemir & Clark, 2007; Vosniadou, 2019; for some examples of long-term longitudinal studies see Blown & Bryce, 2006; Brock & Taber, 2020; Clark, 2006). Hence, even after several years of formal instruction, a substantial proportion of university students continue to fundamentally misunderstand key concepts from e.g., Newtonian mechanics (Caramazza et al., 1981; Clement, 1982b), biology (Dar-Nimrod & Heine, 2011; Shtulman, 2006), physics (Burgoon et al., 2011; Cohen et al., 1983), or mathematics (Clement, 1982a; Graeber et al., 1989; for a recent review on persisting misconceptions in science, see Shtulman & Walker, 2020). To take an illustrative example in mathematics, many middle-schoolers fail to add, subtract, multiply or compare two fractions, or to place simple fractions on number lines (Jordan et al., 2016; Resnick et al., 2016; for similar difficulties in adults, see Post & Harel, 1991; Schneider & Siegler, 2010)—this despite the fact that fractions are typically introduced in 4^th^ grade. Furthermore, many children, adolescents and even adults fail to apprehend that, unlike Integers, fractions form a dense set, i.e., there are infinitely many fractions between any two fractions (Smith et al., 2005; Vamvakoussi & Vosniadou, 2010; for a review see Vosniadou et al., 2008). After years of study, these students thus are still at a loss with properties at the core of the concept of rational number.

Why is concept learning so fallible, and what happens during these long periods of time? Most authors agree that students progress towards a better understanding little by little, in a gradual manner (Carey, 2009; diSessa, 2014; Nussbaum, 1989; Ohlsson, 2009; Özdemir & Clark, 2007; Posner et al., 1982; Thornton, 1997; Vosniadou, 2002). Under this assumption, learning could be slow for several reasons: for instance, the number of incremental steps to complete could be very large, learners may often err in wrong directions instead of progressing towards a more accurate understanding, and/or progress may be fragile. Hence, children may find support for their intuitive (and wrong) conceptions in their everyday experience (Shtulman, 2022), with experience thus acting as a counterforce constantly undoing the progress achieved in class. Alternatively, or perhaps in complement to incremental learning, learners may need to go through discrete leaps of understanding in order to acquire difficult science concepts (Clement, 1989; Gilbert & Watts, 1983)—perhaps suggestively, many theorists of conceptual change (including contenders of gradual learning) have described key learning requirements in terms of qualitative shifts (Carey, 2009; Chi, 2008; Ohlsson, 2009; Posner et al., 1982; Vosniadou et al., 2008). If this second suggestion holds, learning scientific concepts could be particularly long and fallible because the processes bringing about discrete leaps of understanding are themselves extremely fallible: they rarely complete successfully, imposing a bottleneck at key learning steps.

Are scientific concepts sometimes acquired in discrete leaps? In the making of science at least, conceptual progress sometimes *feels* discrete. For instance, the mathematician Henri Poincaré described his astounding discovery of the Fuchsian functions in these terms: “*At the moment when I put my foot on the step, the idea came to me, without anything in my former thoughts seeming to have paved the way for it (…). I did not verify the idea; I should not have had time, (…) but I felt a perfect certainty.*” (Poincaré, 1946). Like Poincaré, many scientists reported episodes where a new understanding occurred to them suddenly, in an unexpected flash. To cite but a few others, Gauss, Kekulé, and Helmholtz recounted such “Eureka experiences” leading to major advances in mathematics, chemistry, or physics (Clement, 1989; Gruber, 1995; Hadamard, 1954; Horvitz, 2002).

Eureka experiences are not reserved to privileged minds. Psychologists have identified a number of tasks, amongst them the famous Gestaltists’ “insight problems”, which often raise experiences similar to Poincaré’s Eureka on Fuchsian functions (Ohlsson, 1984; Webb et al., 2018). Specifically, when participants solve these tasks, they produce the full solution at once rather than elaborating it progressively (Kaplan & Simon, 1990; Maier, 1931); they are not aware that they are approaching the solution, even seconds before solving the problem (Laukkonen et al., 2021; Metcalfe, 1986a, 1986b; Metcalfe & Wiebe, 1987); and moreover, the solution found is immediately perceived as correct and relevant (Danek & Wiley, 2017; Gick & Lockhart, 1995; Laukkonen et al., 2020). Importantly, and unlike the episodes recounted in scientists’ memoires years after the discoveries were made, this research is based on measures collected while the participants were engaged in solving an experimental task, thus establishing the existence of Eureka experiences and their causal relation with the tasks at hand on a firm experimental ground.

While Eureka experiences have now been proven to arise in a range of contexts (e.g., Bowden et al., 2005; Danek et al., 2014; MacGregor & Cunningham, 2008; Webb et al., 2018), still there is little empirical work, if any, testing whether people may experience Eurekas when learning scientific concepts (for a review, see Brock, 2015). Several reports describe episodes where a student displays excitement while formulating a (correct) idea they had never expressed before, a sign that these students may have received a sudden insight (e.g., Blown & Bryce, 2006; Parnafes, 2012; Srivastava & Ramadas, 2013). Besides these case studies, to our knowledge only one study attempted to describe learning-related Eureka experiences at the population level (Liljedahl, 2005), finding reports of such experiences in 68% of students; but this study suffers from methodological limitations, casting doubts about its quantitative results. Yet, the idea that learning proceeds by sudden illuminations followed by rapid progress is common amongst professional teachers (Brock, 2015; Czarnocha & Baker, 2021) and in the general population. Many readers will probably recall episodes where they felt that they suddenly understood a notion: “Now, I get it!”

Our study was undertaken to address two main questions. First, do people generally experience Eureka moments when learning a new scientific concept? Second, if they do experience Eureka moments, is the impression to have received a sudden insight accurate or illusory? Two aspects of Eureka experiences were examined with this second question in mind. First, to assess people’s impression to have gained a new understanding, we tested whether Eureka experiences are associated with genuine learning progress. Second, in a typical Eureka moment, an idea appears to break into consciousness suddenly and unexpectedly—or, to quote again Poincaré’s words, this insight comes “*without anything in [one’s] previous thinking seemingly paving the way for it*”. To probe the veracity of this impression, we tested whether the progresses of Eureka-triggering mechanisms are accessible to reflective introspection. If learners’ first-person report is accurate, these mechanisms should be unconscious; and in particular, their progress should not inform learners’ reflective judgments about their own learning.

To address these questions, we developed a paradigm where participants were taught the mathematical concept of geodesic, which generalizes the common notion of a straight line to straight trajectories drawn on curved surfaces (Spivak, 1979). Participants were given 1 to 7 lessons to learn about the geodesics of the sphere, and were then administered several tasks testing their understanding of geodesics on the sphere and on other surfaces. In addition, they were asked to reflect on their own learning and assess their confidence in their own understanding, and also to report whether they had experienced Eureka episodes during the course of the experiment.

Our analyses tested four predictions. First, before addressing questions about the relation between Eureka experiences and learning, we needed to demonstrate that our paradigm was effective in producing learning. To that aim, we checked whether participants performed better in the post-teaching tests when they had studied more lessons. Second, if concept learning gives rise to Eureka experiences, then participants should report Eureka experiences, and these reports should be modulated by our experimental manipulation (number of lessons studied): an indication that the experiences reported are induced by the teaching phase, not by e.g., personality traits of individual participants or by the general context of the experiment. Third, if Eureka experiences reflect genuine learning progresses, participants who report Eurekas should achieve better performance in the post-teaching tests. Fourth, we compared the learning achievements associated to Eureka experiences vs. to participants’ reflective judgments of confidence in their own understanding. If Eureka-triggering mechanisms are inaccessible to reflective introspection, Eureka experiences should be uniquely associated to some learning achievements, after factoring out variations in participants’ judgments of confidence. Note that the reverse relation may also be true: perhaps judgments of confidence also relate to specific learning achievements, independently from the occurrence of Eureka experiences. Observing such a pattern of unique associations would indicate that the mechanisms triggering Eurekas and informing reflective confidence are at least partially dissociated, thus suggesting that the concept learning mechanisms that are responsible for Eureka experiences operate unconsciously.

## METHODS

Our experiment comprised four phases. In the first, *inclusion* phase, we administered two tests to select participants who had a good understanding of elementary planar geometry, but were not yet able to identify “straight lines”^1^ (geodesics) on the sphere. Next, in the *teaching* phase, participants were presented with lessons about the “straight lines” of the sphere. The third phase, *test* phase, assessed participants’ understanding of the concept of straight line, generalized to curved surfaces. Lastly, in the fourth or *Eureka* phase, participants were asked if they had experienced Eureka moments during the course of the experiment.

As explained above, in our analyses we aimed to test whether Eureka experiences were associated with genuine learning, and also whether Eureka experiences and confidence judgments were associated to the same kinds of learning. Our *test* phase was thus designed to provide a systematic assessment of participants’ understanding of straight lines, with tasks probing various abilities entering into the possession of a concept. Thus, we included both identification tasks where participants needed to recognize instances of straight lines (i.e., tasks assessing the extension of their concept of straight line), as well as tasks testing their ability to draw inferences about straight lines (i.e., tasks assessing the inferential role of their concept; for an argument that both extension and inferential role participate to the characterization of concepts, see Carey, 2009). For both identification and inference tasks, we furthermore varied the domain of application of the concept: either the sphere (the domain of application covered in the lessons), or non-sphere surfaces that were not mentioned in the lessons (on contextual effects and lack of transfer in science concept learning, see e.g., Brock & Taber, 2020). Finally, in our identification tasks we systematically crossed two variables: straightness and planarity. Indeed, based on a study testing intuitions in spherical geometry (Izard et al., 2011), we hypothesized that people would spontaneously be biased to identify straight lines with planar cuts, at least on the sphere (for direct evidence supporting this hypothesis across many surfaces, see Barot, 2022). The systematic manipulation of planarity alongside straightness thus allowed us to more finely probe the nature of the geometric properties defining “straight lines” for our participants. Specifically, if participants rely on their spontaneous intuitions, they should respond on the basis of planarity; whereas if they apply the criteria given in the lessons, their responses should be driven by straightness.

These manipulations yielded nine different test conditions: 3 test conditions assessing participants’ identification of straight lines on the sphere (non-planar straight lines do not exist on the sphere so the two variables of straightness and planarity could not be fully crossed), 4 test conditions assessing participants’ identification of straight lines on various surfaces (fully crossing straightness and planarity), 1 test condition assessing inferences about straight lines on the sphere, and 1 test condition assessing inferences about straight lines on various surfaces.

### Participants

Participants were recruited from a mailing list of volunteers from the greater Paris area or by word of mouth, with three inclusion criteria: being aged between 18 and 50 years, having good corrected vision, and being fluent in French. A total of 69 persons were tested, but the data of 13 participants were excluded from analyses because of their performance on inclusion tests (5 participants for poor performance in planar geometry, 3 participants for good performance in spherical geometry) and/or because of an experimenter error (6).

The final sample included 56 participants (40 females, age 18–43 years, Mean = 25.5 years, exact age missing for 10 participants)^2^. All of them had attended high school. In France, students can choose to specialize in the humanities and quit studying mathematics after completing 10th grade. In our sample, some participants had thus received education in mathematics only until 10th grade, while others had received up to 7 additional years of mathematics education (average number of years of education in mathematics after 10th grade: 3.9 years).

The study was conducted according to the ethical standards of Helsinki’s declaration. Participants provided written informed consent before starting the experiment. The experiment lasted 60 to 90 minutes and all participants received a 15€ compensation. To support people’s motivation to learn, a 50€ bonus was awarded to the participant who reached the highest score in each teaching condition.

### Material and Procedure

Table 1 describes the content of the four phases of the experiment (inclusion phase, teaching phase, test phase, and Eureka phase)^3^.

**Table 1.** Tasks administered to the participants in the four phases of the experiment.

**Inclusion phase**	**Teaching phase**	**Test phase**	**Eureka phase**
Planar geometry test	Introduction: great circles	Confidence judgment (1)	Eureka report
Straight lines identification on spheres	1, 3, 5, or 7 lessons about straight lines on the sphere	Straight lines identification on spheres	
		Straight lines identification on various surfaces	
		Confidence judgment (2)	
		Reasoning about straight lines on the sphere and on other surfaces	
		Confidence judgment (3)	

#### Inclusion Phase.

In the first phase of the experiment, participants were administered two tests assessing respectively their understanding of planar and spherical geometry. They were included in the main analyses if they had a typical (and good) understanding of elementary planar geometry but were not able to identify straight lines on spheres.

##### Planar Geometry Test.

This test was adapted from Izard et al. (2011; planar geometry condition, questions 1–20). Participants were first introduced to a planar surface, extending indefinitely, on which points and straight lines could be drawn. Straight lines were described as lines that never turn, neither on the left nor on the right, and that continue straight ahead indefinitely. After this introduction, participants were asked a series of twenty illustrated questions about the properties of straight lines on this infinite plane. For example, in one of the trials, they were presented with a figure showing a straight line and a point and asked whether it is possible to draw a new line that goes through the point and does not intersect the first line. Questions were presented both in writing and orally through an audio recording, and participants ticked their answers (yes or no) on a response sheet. Participants were included if they made no more than 3 errors—pilot work indicated that more than 90% of geometry educated adults should pass this criterion.

##### Straight Lines Identification on Spheres.

**Figure 1.**
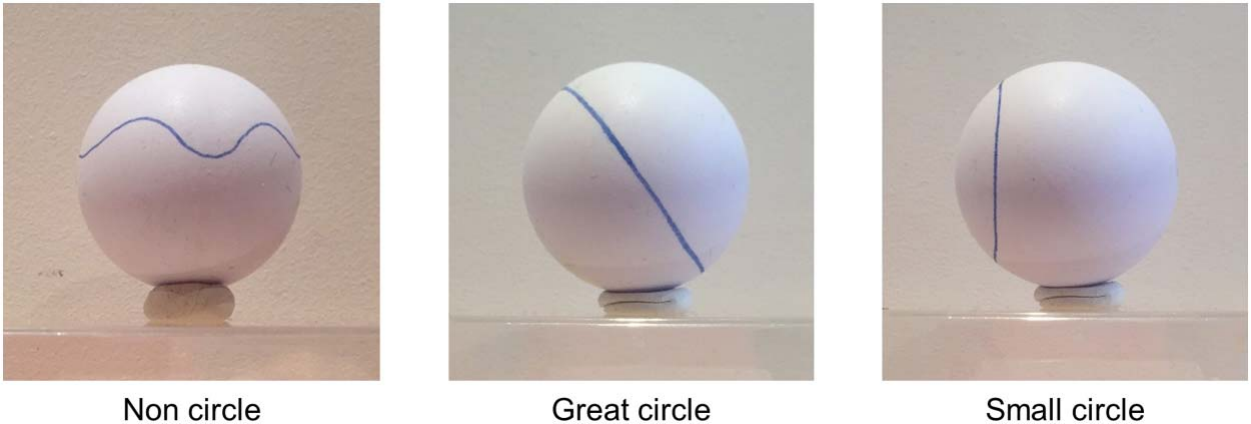
Example stimuli from the three conditions of the spheres straight lines identification task.

In each trial, participants were presented with a photograph of a sphere (a table tennis ball) with a line drawn on it, and were asked to indicate whether the line was “straight” or not. Three types of trials were presented (Figure 1): noncircles (non-straight; e.g., wavy line, line looping and crossing itself to form an 8 figure), great circles (straight), and small circles (non-straight, but typically judged to be straight by most adults). Each category counted 4 trials, for a total of 12 trials presented in a randomized order.

Participants responded by pressing the ‘O’ key for yes (in French: ‘oui’) or the ‘N’ key for no (‘non’). They were included if they made at least two mistakes on the small circle trials (i.e., they incorrectly judged small circles to be straight lines) or if they made at least two mistakes on the great circle trials (i.e., they incorrectly judged that great circles are not straight).

#### Teaching Phase.

In the second phase, participants were taught how to generalize the concept of “straight line” to the sphere. They were first given a one-page introduction defining the “great circles” of a sphere as circles that have the same radius as the sphere on which they are drawn. This introduction also provided illustrations of great circles drawn at different orientations. Then, participants studied 1, 3, 5, or 7 lessons about straight lines in spherical geometry. The lessons used simple physical models to explain why great circles correspond to straight lines on the sphere, but smaller circles do not. These models were: rolling a toy car on a ball (the car can be rolled along a great circle, but not along a small circle; 2 lessons), applying scotch tape on a ball (the tape wrinkles if applied along a small circle, but remains smooth if applied along a great circle; 2 lessons), pinning a rubber band on a ball (the rubber band naturally aligns with a great circle; 2 lessons), and flight routes (flight routes seem curved when mapped on a planisphere, but prove to be the shortest route and a portion of a great circle when mapped on a globe; 1 lesson). For each teaching condition, different orders of presentation were created to ensure that each lesson appeared in each position, and that a given lesson was not always followed or preceded by the same lesson.

A translated version of the teaching materials can be found on the Github repository of the project: https://github.com/charlusb/Analyses_Eurekamaths.

#### Test Phase.

At the end of the teaching phase, the first experimenter left the room and was replaced by a second experimenter, who was blind to the teaching condition assigned to the participants. This second experimenter administered three tasks: In the first task, participants judged whether lines drawn on a sphere were straight or not; in the second task they judged whether lines drawn on different surfaces were straight or not; and in the third task they were asked to draw inferences about the geometric properties of straight lines on the sphere and on other surfaces. We also measured participants’ confidence in their own understanding of straight lines at different time points during the test phase.

##### Straight Lines Identification on Spheres.

This task was identical to the spherical geometry inclusion task.

##### Straight Lines Identification on Various Surfaces.

**Figure 2.**
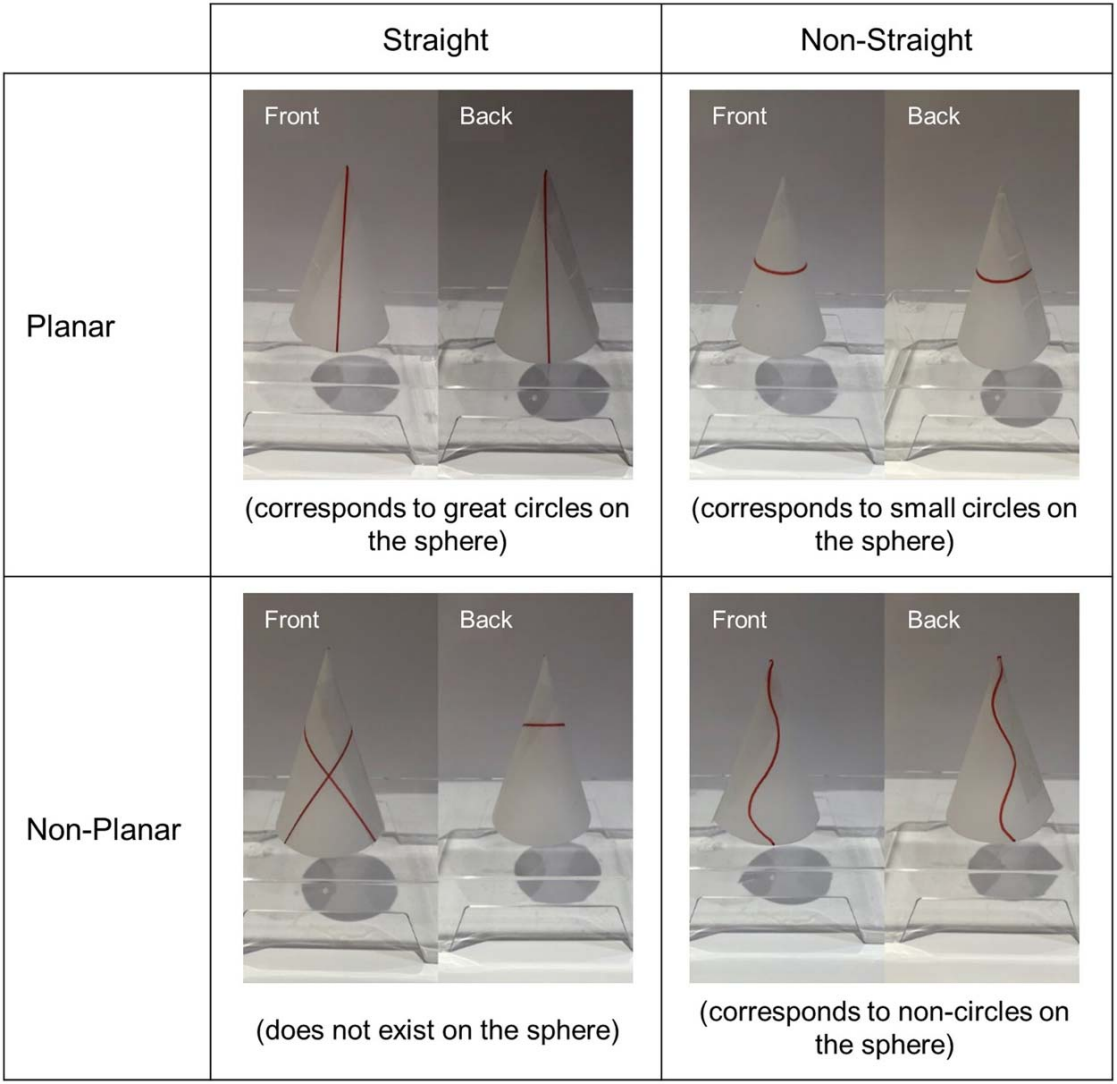
Example stimuli from the four conditions in the various surfaces straight lines identification task. Each trial presented two photographs showing a front and a back view of a surface and labeled as such. Three of the conditions correspond to lines that can be realized on the sphere (corresponding to great circles, small circles, and non-circles) while the last one (straight non-planar) does not exist on the sphere.

Participants were presented with lines drawn on four different surfaces: cone (8 trials), cylinder (6 trials), cube (8 trials), and torus (4 trials). Each trial displayed photographs of the front and back view of a surface on which a line had been drawn (Figure 2). Participants were asked to judge whether the line presented was straight or not and indicated their answer by keypress. As explained above, we suspected that lines corresponding to the intersection of a surface with a plane would intuitively look straight, as they do on the sphere (on the sphere, planar intersections correspond to circles). Our task thus crossed the two variables of straightness (straight, not straight) and planarity (planar, non-planar): 4 trials presented non-planar non-straight lines (corresponding to noncircle lines on the sphere), 3 trials presented planar straight lines (corresponding to great circles on the sphere), 10 trials presented planar non-straight lines (corresponding to small circles on the sphere), and 9 trials presented non-planar straight lines (there are no corresponding examples on the sphere, but such lines can exist on other surfaces). Trials were presented in random order.

##### Reasoning About Straight Lines on the Sphere and on Other Surfaces.

This task consisted of a list of mathematical assertions, which participants judged to be true or false: eight assertions about the straight lines of the sphere, followed by eight assertions about straight lines on arbitrary surfaces (Table 2). The assertions were presented in a fixed order, on paper.

**Table 2.** Assertions of the reasoning task. Assertions 1–8 focus on the sphere, while assertions 9–16 invite participants to think about various surfaces. Participants were provided with the following definitions on the top of the page: “Two straight lines are parallel if they never intersect. Two straight lines are perpendicular if they intersect at a right angle”. All the material presented here is translated from French.

**Assertion**	**Correct answer**
1. On a sphere, given two straight lines, one can draw a straight line that intersects the first one but not the second one.	False
2. On a sphere, one can draw two straight lines that get closer to each other.	True
3. On a sphere, there is an infinity of lines perpendicular to a given line (not necessarily at the same point).	True
4. On a sphere, one can draw two straight lines that never intersect.	False
5. On a sphere, it is possible to draw two straight lines that are perpendicular.	True
6. On a sphere, two distinct straight lines always have two points of intersection.	True
7. On a sphere, it is possible to draw a straight line that is parallel to a first straight line and goes through a given point.	False
8. On a sphere, two straight lines can be drawn at a constant distance from each other.	False
9. There is a surface on which there is always one single straight line that is parallel to a first straight line and that goes through a given point.	True
10. There is a surface on which it is never possible to draw a straight line that is parallel to a first straight line and that goes through a given point.	True
11. There is a surface on which a straight line can go several times through the same point (intersecting itself).	True
12. There is a surface on which two straight lines can be drawn at a constant distance from each other.	True
13. There is a surface on which it is possible to draw several straight lines going through two given points.	True
14. There is a surface on which two straight lines are always intersecting.	True
15. There is a surface on which it is not possible to draw two perpendicular lines.	False
16. There is a surface on which it is not possible to draw two straight lines that intersect.	False

Participants were given written definitions for the terms ‘parallel’ and ‘perpendicular’, which appeared in some of the assertions. They answered by ticking one of four response options for each assertion: ‘true - certain’, ‘true - uncertain’, ‘false - uncertain’, and ‘false - certain’. These four options were introduced to help participants decide when they were unsure, but we only analyzed the valence of the response (true/false).

##### Confidence Judgments.

At three different times, participants indicated how much they felt they understood the notion of straight line, on a scale graduated from 0 to 10. The first measurement of confidence was taken at the end of the teaching phase, the second measurement after the various surfaces straight lines identification task, and the third measurement after the reasoning task. One participant in the 1-lesson group inadvertently failed to answer the third confidence question.

#### Eureka Phase.

The final part of the experiment, which was administered by the first experimenter, aimed at measuring whether participants had experienced Eurekas during the course of the experimental session. Participants were first given a description of the sensations associated with Eureka experiences (adapted from Jung-Beeman et al., 2004): episodes where a new understanding arises suddenly and unexpectedly, and is associated with a feeling of certainty. Participants indicated whether they experienced such episodes at some point during the experiment (yes or no)—this answer was used as our measure of Eureka report.

At the end of the session, participants were presented with vignettes illustrating the different phases of the experiment, so that they could indicate when exactly they had experienced Eurekas. A few participants were also asked to describe the insights that occurred to them in these occasions: they all reported insights related to the concept of straight lines on non-planar surfaces.

### Analyses

We conducted analyses to address four questions: (i) whether the participants studying more lessons performed better in post-teaching tests and (ii) were more likely to report experiencing Eurekas, (iii) whether the participants reporting Eurekas showed better learning, as assessed by better performance in post-teaching tests, and (iv) whether Eureka reports and reflective judgments of confidence were associated to similar or different profiles of performance across the post-teaching tests. Analyses were conducted in R using the packages afex and emmeans (Lenth, 2022; Singmann et al., 2021). For repeated measures analyses, we used the function mixed in package afex^4^ and included a random intercept for participant^5^. For non-repeated measures analyses, we used the standard glm function. The *α* level was set at .05. Significant interactions involving a numerical variable (e.g., number of lessons) were explored by computing linear trends by condition (function emtrends in emmeans). Significant interactions between categorical variables were explored by computing contrasts by condition (function emmeans^6^). Holm’s procedure was used to control for multiple comparisons when exploring interactions.

Analyses scripts are available on the Github repository of the project: https://github.com/charlusb/Analyses_Eurekamaths.

#### Effect of the Number of Lessons on Test Phase Performance.

First, to assess whether our paradigm effectively induced learning, we tested whether participants’ objective performance in post-teaching tests varied as a function of the number of lessons studied. For the sake of simplicity, and to reduce the number of statistical tests performed, all the tasks of the test phase were analyzed in a single analysis. Accuracy was entered in a logistic mixed model analysis, with a random intercept for participant, a categorical variable for test condition (total of 9 test conditions corresponding to 3 test conditions in the sphere straight line identification task: non-circle lines, great circles, small circles; 4 test conditions in the various surfaces straight line identification task: non-planar non-straight, planar straight, planar non-straight, non-planar straight; and 2 test conditions in the reasoning task: sphere, surfaces), numerical variables for teaching condition (number of lessons studied) and education in mathematics (number of years studying mathematics), as well as interactions between test condition and number of lessons, and between test condition and education in mathematics (formula: Accuracy ∼ TestCondition * NumberLessons + TestCondition * MathEducation + (1|Participant)). If our manipulation was successful, we expected this analysis to yield a main effect of the number of lessons, and/or a significant interaction between test condition and number of lessons.

#### Effect of the Number of Lessons on Eureka Experiences.

To analyze whether studying a mathematical concept triggers Eureka experiences, we tested whether Eureka reports were modulated by the experimental manipulation introduced in the teaching phase, i.e., the number of lessons presented. Eureka reports were entered in a logistic regression with two numerical variables for the number of lessons studied and participants’ education in mathematics (Eureka ∼ NumberLessons + MathEducation). An effect of the number of lessons in this analysis would indicate that the Eureka reports observed in our study are causally related to the teaching phase, and not simply to the general context of the experiment. Furthermore, it would allow us to exclude explanations based exclusively on inter-individual variations in e.g., personality traits or education in mathematics.

#### Relation between Eureka Experiences and Test Phase Performance.

We next tested whether the participants who experienced Eurekas reached a better level of understanding, as indicated by better performance in the test phase. To do so, we used a logistic mixed model on accuracy with a random intercept for participant and fixed effects for test condition (categorical variable with 9 levels as above), Eureka report (binary variable indicating whether the participant reported experiencing any Eurekas during the course of the experiment or not), and an interaction between test condition and Eureka report (Accuracy ∼ TestCondition * Eureka + (1|Participant)). We then conducted a second version of this analysis with additional variables for number of lessons and years of education in mathematics and their interaction with test condition (Accuracy ∼ TestCondition * Eureka + TestCondition * NumberLessons + TestCondition * MathEducation + (1|Participant)). This second analysis is more conservative and detects relations between Eureka experiences and performance that cannot be explained through the influence of the teaching condition or participants’ education in mathematics. However, the first analysis without covariates is potentially informative as well: if teaching condition and education in mathematics constitute the main source of variance between participants, introducing these variables as covariates may drastically reduce variability and render relations between Eureka experiences and performance impossible to detect.

#### Relation between Eureka Experiences and Confidence.

The last series of analyses aimed at testing whether confidence in one’s own understanding and Eureka experiences reflect similar or different learning processes. To approach this question, we first tested whether Eureka reports and confidence judgments were correlated to each other. Pairwise comparisons were conducted between four measures: the three ratings of confidence collected throughout the test phase, and the Eureka report collected at the end of the experiment. For each comparison, we conducted two Spearman correlation analyses, first without covariates, and second with number of lessons and years of education in mathematics as covariates. In each version of the analysis, *p*-values were corrected for multiple comparisons using Holm’s procedure.

Second, we tested whether Eureka reports and confidence ratings were related to learning the same aspects of the target mathematical concept. To do so, we conducted a logistic mixed model analysis on test phase accuracy with a random intercept for participant and fixed effects for test condition (categorical variable with 9 levels as above), Eureka report, confidence ratings, as well as interactions of Eureka report and confidence ratings with test condition. Again, two versions of this analysis were conducted: one without variables accounting for number of lessons and years of education in mathematics (Accuracy ∼ TestCondition * Eureka + TestCondition * Confidence + (1|Participant)), and one including these variables and their interaction with test condition as fixed effects (Accuracy ∼ TestCondition * Eureka + TestCondition * Confidence + TestCondition * NumberLessons + TestCondition * MathEducation + (1|Participant)). Since the correlation analysis found all three ratings of confidence to be highly correlated, here we used the mean of participants’ three ratings. Observing significant interactions of test condition with Eureka report and confidence rating in this analysis would indicate that Eureka experiences and reflective judgments of confidence are related to different learning achievements, and would thus suggest that the processes giving rise to Eureka experiences are inaccessible to reflective introspection.

## RESULTS

### Effect of the Number of Lessons on Test Phase Performance

**Figure 3.**
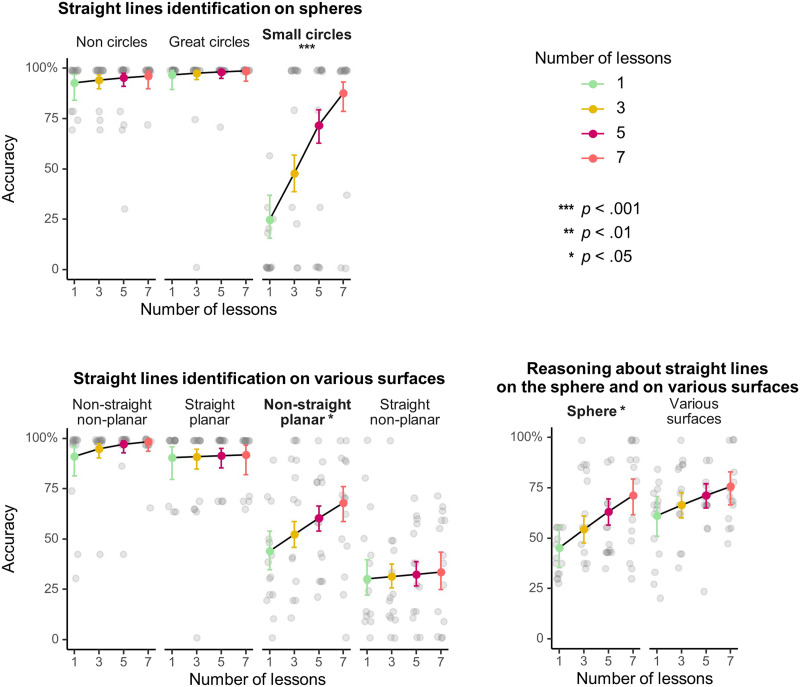
Effect of the number of lessons on accuracy in each test condition. Colored dots and plain lines display the predictions of the logistic mixed model. Error bars represent 95% CI. Transparent grey dots show the performance of individual participants, corrected for years of mathematics education. *P*-values from a post-hoc exploration of the linear trends by test condition, holm-corrected for multiple comparisons. Test conditions showing a significant effect of number of lessons are highlighted in **bold**.

Performance varied across test conditions (main effect of test condition, *p* < .001, Table 3), ranging from 33.1% (straight line identification: straight non-planar lines on various surfaces) to 97.3% (straight line identification: great circles on spheres). In line with our expectations, the teaching phase manipulation had an impact on participants’ objective performance on post-teaching tests, as attested by both a main effect of the number of lessons and a significant interaction between test condition and number of lessons. To explore the effect of the number of lessons in each test condition, we computed linear trends by number of lessons for each test condition (Figure 3). We found a positive effect of the number of lessons on participants’ ability to categorize small circles on spheres as non-straight (Estimated Trend (ET) = 0.51, 95% CI = [0.27, 0.75], *p*_*corr*_ < .001), as well as, more generally, on their ability to categorize planar non-straight lines on various surfaces as non-straight (same type of line as small circles on spheres; ET = 0.16, 95% CI = [0.01, 0.31], *p*_*corr*_ = .017). Participants who studied more lessons also performed better when asked to draw inferences about the properties of straight lines on the sphere in the reasoning test (ET = 0.18, 95% CI = [0.02, 0.35], *p*_*corr*_ = .014). Estimated trends were non-significant in the other test conditions (*p*_*corr*_’s > .28; detailed results are provided in the Supplementary Online Material).

**Table 3.** Logistic mixed model analysis of the effect of number of lessons on test phase accuracy.

	df	*χ* ^2^	*p*
**Test condition**	**8**	**696.3**	**< .001**
**Number of lessons**	**1**	**11.9**	**< .001**
Education in Mathematics	1	2.7	.097
Test condition * Number of lessons	**8**	**36.2**	**< .001**
Test condition * Education in mathematics	8	11.0	.20

Results of the logistic mixed model analysis of the effect of the number of lessons on test phase accuracy. Shown here are likelihood ratio tests comparing the full model to restricted models lacking one of the predictors. Full model formula: Accuracy ∼ TestCondition * NumberLessons + TestCondition * MathEducation + (1|Participant); Full model fit: LogLik = −1536.2, Random intercept (participant): variance = 0.36. Significant effects are highlighted in **bold**.

### Effect of the Number of Lessons on Eureka Experiences

**Figure 4.**
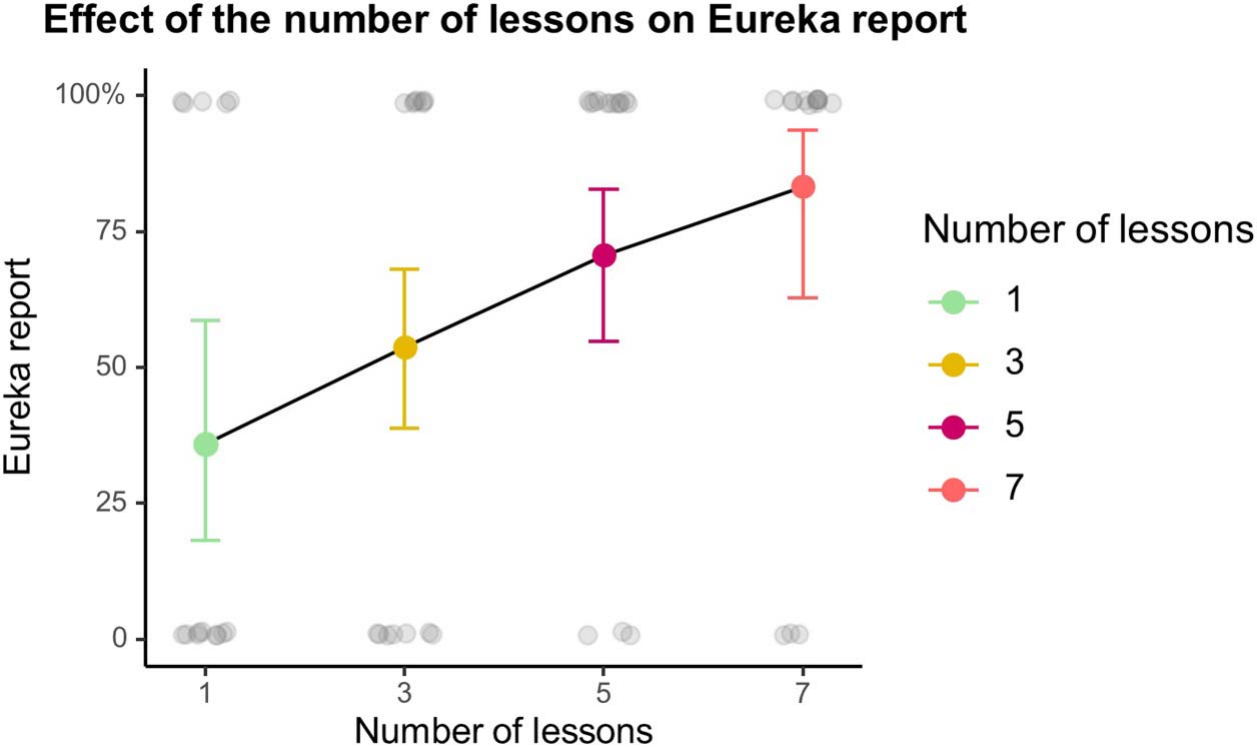
Effect of the number of lessons on Eureka reports. Colored dots and plain lines display the predictions of the logistic regression model. Error bars represent 95% CI. Transparent grey dots show the responses of individual participants, corrected for years of mathematics education.

Thirty-four of our fifty-six participants reported experiencing a Eureka moment (61%). A logistic regression analysis (Table 4; Figure 4) revealed that Eureka reports were influenced by the number of lessons the participants were given to study but not by their education in mathematics.

**Table 4.** Logistic regression analysis of the effect of number of lessons on Eureka report.

	*χ* ^2^	df	*p*
**Number of lessons**	**7.8**	**1**	**.005**
Education in mathematics	0.7	1	.42

Results of the logistic regression analyzing the effect of the number of lessons on Eureka reports. Shown here are likelihood ratio tests comparing the full model to restricted models lacking one of the predictors. Full model formula: Eureka ∼ NumberLessons + MathEducation; Full model fit: LogLik = −33.4. Significant effects are highlighted in **bold**.

### Relation between Eureka Experiences and Test Phase Performance

The relation between Eureka report and test phase performance was assessed in two mixed model analyses with and without covariates accounting for number of lessons and years of education in mathematics. Both analyses identified a significant interaction between a variable measuring whether participants reported Eurekas and the test condition variable (Table 5), indicating that participants who did vs. did not report experiencing Eurekas differed in their performance on some of the test conditions. Exploring these interactions revealed that participants who experienced Eurekas were more likely to identify lines that are straight despite being not planar in the various surfaces straight line identification task—the type of line that does not exist on the sphere (model without covariates: Estimated Contrast (EC) = 1.06, 95% CI = [0.27, 1.86], *p*_*corr*_ = .002; model with covariates: EC = 1.22, 95% CI = [0.39, 2.05], *p*_*corr*_ < .001). In addition, there was a significant effect of Eureka experience on participants’ accuracy at rejecting small circles on the sphere, but only in the model that did not account for number of lessons or years of education in mathematics (model without covariates: EC = 0.96, 95% CI = [0.00, 1.92], *p*_*corr*_ = .043; model with covariates: EC = 0.39, 95% CI = [−0.67, 1.45], *p*_*corr*_ = 1.00). In all the other test conditions, the linear trends did not reach significance (model without covariates: *p*_*corr*_’s = 1.0; model with covariates: *p*_*corr*_’s > .62; see Supplementary Online Material for detailed statistics by test condition).

**Table 5.** Logistic mixed model analysis of the relation between Eureka report and test phase accuracy.

	No covariate analysis			Covariates analysis		
	df	*χ* ^2^	*p*	df	*χ* ^2^	*p*
**Test condition**	**8**	**724.3**	**< .001**	**8**	**719.7**	**< .001**
Eureka report	1	0.0	.98	1	1.0	.33
**Number of lessons**				**1**	**12.3**	**< .001**
Education in mathematics				1	2.2	.14
Test condition * Eureka report	**8**	**27.1**	**< .001**	**8**	**34.4**	**< .001**
Test condition * Number of lessons				**8**	**43.0**	**< .001**
Test condition * Education in mathematics				8	12.0	.15

Results of the mixed model analyses of the relation between Eureka report and test phase accuracy. Shown here are likelihood ratio tests comparing the full model to restricted models lacking one of the predictors. Left: analysis without covariates. Full model formula: Accuracy ∼ TestCondition * Eureka + (1|Participant); Full model fit: LogLik = −1551.5, Random intercept (participant): variance = 0.45. Right: analysis with covariates for years of education in mathematics and number of lessons. Full model formula: Accuracy ∼ TestCondition * NumberLessons + TestCondition * MathEducation + TestCondition * Eureka + (1|Participant); Full model fit: LogLik −1518.5, Random intercept (participant): variance = 0.36. Significant effects are highlighted in **bold**.

### Relation between Eureka Experiences and Confidence

#### Correlation Tests.

Tests of the correlations between participants’ three ratings of confidence and their report of Eureka experiences were conducted twice, once without covariates, and once with number of lessons and years of education in mathematics as covariates. In the two versions of the analysis, the three ratings of confidence were strongly correlated to each other (Table 6). In contrast, ratings of confidence did not correlate with Eureka reports.

**Table 6.** Correlation table for confidence ratings and Eureka report.

	Confidence 1	Confidence 2	Confidence 3	Eureka
Confidence 1	X	***ρ*(54) = .59 *p* < .001**	***ρ*(53) = .60 *p* < .001**	*ρ*(54) = .27 *p* = .13
Confidence 2	***ρ*(52) = .57 *p* < .001**	X	***ρ*(53) = .85 *p* < .001**	*ρ*(54) = .19 *p* = .33
Confidence 3	***ρ*(51) = .60 *p* < .001**	***ρ*(51) = .85 *p* < .001**	X	*ρ*(53) = .17 *p* = .33
Eureka	*ρ*(52) = .15 *p* = .81	*ρ*(52) = .14 *p* = .81	*ρ*(51) = .15 *p* = .81	X

Spearman’s *ρ* coefficients and *p*-values for pairwise correlation tests. Above diagonal: without covariates, below: with number of lessons and years of education in mathematics as covariates. Significant correlations are highlighted in **bold**. All *p*-values were corrected for multiple comparisons using Holm’s method (applied separately for the analyses with and without covariates). Note that the third rating of confidence was missing for one participant in the 1-lesson group, hence the difference in degrees of freedom. Confidence 1: measured just after participants completed the teaching phase; Confidence 2: measured after the various surfaces straight lines identification task; Confidence 3: measured after the reasoning task.

#### Relation between Eureka Experiences, Confidence and Test Phase Performance.

**Figure 5.**
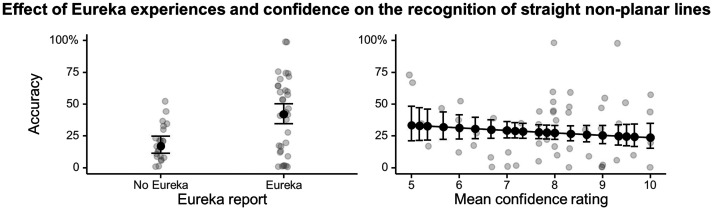
Relation between test phase performance, Eureka report and confidence judgments in the condition of the straight lines identification task that has no equivalent on the sphere (straight non-planar lines). (A) Predicted performance for participants who did vs. did not report Eureka experiences, and individual participants’ performance corrected for years of mathematics education, number of lessons, and confidence level. (B) Predicted performance for participants who reported different levels of confidence, and individual participants’ performance corrected for years of mathematics education, number of lessons and Eureka report. Error bars are 95% confidence intervals.

Lastly, we tested whether Eureka experiences and reflective judgments of confidence were related to learning the same aspects of the concept of generalized straight lines. Again, we conducted two mixed model analyses, one with and one without covariates accounting for number of lessons and years of education in mathematics. The two analyses yielded significant interactions between Eureka report and test condition, as well as between confidence and test condition (Table 7). The analysis with covariates also identified a significant main effect of confidence. Exploring the interaction between Eureka and test condition revealed again a positive relation between Eureka report and performance in the condition from the various surfaces straight lines identification task that has no equivalent on the sphere (non-planar straight lines), indicating that this relation arises independently of participants’ confidence in their own understanding (model without covariates: EC = 1.15, 95% CI = [0.32, 1.97], *p*_*corr*_ = .001; model with covariates EC = 1.26, 95% CI = [0.43, 2.10], *p_corr_* < .001; Figure 5). None of the other test conditions showed a significant association of performance with Eureka report (model without covariates: *p*_*corr*_’s > .21; model with covariates: *p*_*corr*_’s > .67). Exploring the interaction between test condition and confidence failed to identify a specific association between confidence and performance in any of our single test conditions (model without covariates: *p*_*corr*_’s > .09; model with covariates: *p*_*corr*_’s > .30). Detailed statistics of the effects of Eureka report or confidence in each test condition are provided in the Supplementary Online Material.

**Table 7.** Logistic mixed model analysis of the relation between Eureka report, confidence and test phase accuracy.

	No covariate analysis			Covariates analysis		
	df	*χ* ^2^	*p*	df	*χ* ^2^	*p*
**Test condition**	**8**	**740.5**	**< .001**	**8**	**736.8**	**< .001**
Eureka report	1	0.0	.93	1	1.4	.24
**Confidence**	1	1.8	.18	**1**	**4.3**	**.039**
**Number of lessons**				**1**	**12.0**	**< .001**
Education in mathematics				1	3.0	.085
Test condition * Eureka report	**8**	**26.1**	**.001**	**8**	**34.2**	**< .001**
Test condition * Confidence	**8**	**33.0**	**< .001**	**8**	**25.9**	**.001**
Test condition * Number of lessons				**8**	**36.1**	**< .001**
Test condition * Education in mathematics				8	10.6	.22

Results of the mixed model analyses of the relation between Eureka report, confidence and test phase accuracy. Shown here are likelihood ratio tests comparing the full model to restricted models lacking one of the predictors. Left: model without covariates. Full model formula: Accuracy ∼ TestCondition * Confidence + TestCondition * Eureka + (1|Participant); Full model fit: LogLik = −1534.9, Random intercept (participant): variance = 0.45. Right: model with covariates for number of lessons and education in mathematics. Full model formula: Accuracy ∼ TestCondition * NumberLessons + TestCondition * MathEducation + TestCondition * Confidence + TestCondition * Eureka + (1|Participant); Full model fit: LogLik = −1505.5, Random intercept (participant): variance = 0.36. Significant effects are highlighted in **bold**.

## DISCUSSION

Our study had four goals. First, as a prerequisite to our other aims, we needed to conceive a one-session, lab-based paradigm that effectively induces learning in mathematics. Second, using this paradigm, we asked whether learning a mathematical concept triggers Eureka experiences: episodes where a new understanding arises in a flash, suddenly and unexpectedly, accompanied with a feeling of certainty. Third, we tested whether these Eureka experiences were reliable, in a first sense: that is, whether experiencing Eurekas was associated with greater learning achievements. Fourth, we probed the reliability of Eureka experiences in a second sense: in the sense that an insight broke into consciousness suddenly—and thus any learning that happened beforehand must have been unconscious. To get at this question, we analyzed whether reports of Eureka experiences and reflective judgments of confidence reflected the acquisition of the same or different aspects of the target concept. If the learning mechanisms leading to sudden flashes of insight are unconscious, we reasoned, they should not contribute to inform participants’ reflective judgments of confidence, and therefore some learning achievements should remain associated with Eureka experiences after factoring out variations in confidence. Below we present our findings and conclusions for each of these four questions.

### Learning About Geodesics

Participants were invited to study one to seven lessons introducing a mathematical concept: the concept of geodesic, generalizing the common notion of a planar straight line to straight trajectories drawn on curved surfaces. All lessons focused on the sphere and appealed to simple physical models to explain why great circles drawn on spheres correspond to lines that are straight, while small circles do not—which is counterintuitive. The models were meant to help participants ground their understanding of “straightness” on physical intuitions: for instance, a line is straight if, along this line, one can smoothly apply a band of scotch tape, or roll a toy car without forcing it to turn. Following this teaching phase, we assessed participants’ learning in a series of tasks probing key conceptual abilities: the ability to identify straight lines, both within the domain of application covered in the lessons (on the sphere), and in new contexts (non-sphere surfaces); and the ability to draw inferences about straight lines, again manipulating the domain of application (sphere vs. various surfaces). Furthermore, our identification tasks systematically crossed line straightness against planarity (whether the line is the result of a planar cut of the surface), as this property strongly influences judgments of straightness in naïve participants (Barot, 2022). Manipulating planarity thus allowed us to assess whether participants succeeded in defining straight lines according to the criteria presented in the lessons, even when doing so leads to a counterintuitive answer.

Our results provide evidence that our teaching phase was effective, and participants learned: reading more lessons led to better performance in several of our post-teaching test conditions. Furthermore, participants’ post-test performance showed two characteristic signatures of conceptual learning. First, learning was difficult, as indicated by positive linear effects of the number of lessons on test performance in several of our test conditions. These effects show that learning was protracted, as it was not completed after studying the first lesson. With more lessons, participants may have benefitted from the repeated presentation of the same mathematical information (great circles are straight, small circles are not straight), from the presentation of diverse physical models to ground the concept of “straightness”, and/or simply from a greater incubation delay. Strikingly, these effects arose even in a test condition where participants simply needed to recall information presented in the lessons, i.e., when judging that small circles drawn on a sphere are not straight. As a second signature of conceptual learning, the content learned was inferentially rich: participants did not only memorize the information presented in the lessons but were also able to draw inferences from this information. Indeed, participants who received more lessons to study had a better performance in a test condition where they needed to generalize the notion of straight line to various surfaces, beyond the example of the sphere (condition presenting non-straight planar lines drawn on non-sphere surfaces, equivalent to small circles drawn on spheres), and in a condition where they needed to reason about the properties of straight lines on the sphere.

In our reasoning task, in particular, we found that the teaching phase enabled participants to draw non trivial inferences about the properties of straight lines in spherical geometry, and notably to realize that two straight lines drawn on a sphere can never be parallel: they necessarily cross. This property is highly counterintuitive for geometry-educated people—as well as for people without any formal education in geometry (Izard et al., 2011). When tested on a questionnaire that inspired our planar geometry inclusion test, pretty much all U.S. adults, French teenagers, or Mundurucu people from the Amazon judged that it was possible to find parallel lines on the plane and also on the sphere (agreement on the existence of parallel lines on the sphere was above 90% in all groups). These widely shared and strongly entrenched intuitions about parallel lines resonate with the History of Mathematics. During nearly 2000 years, mathematicians attempted to demonstrate Euclid’s Fifth Postulate on parallel lines from the other simpler postulates and axioms; until Gauss, Bolyai and Lobachevsky finally realized that this enterprise had started on wrong premises, and that it is possible to define geometries that are perfectly coherent with the simpler axioms but nonetheless violate the postulate on parallels (Greenberg, 2007). The geometry defined by great circles on the sphere is an example of one such coherent “non-Euclidean” geometry. In that context, it is impressive to observe that a considerable proportion of our participants questioned or even refuted the existence of parallel lines on the sphere (45.5% of negative responses for two assertions of the reasoning test claiming that parallel lines exist on the sphere). Again, the teaching phase played a role in this understanding (linear trend of number of lessons for these two assertions, ET = 0.53, 95% CI = [0.08, 0.98], *p* = .020, controlling for years of education in mathematics).

In other test conditions, we did not observe any impact of the teaching phase on performance. Some of these test conditions were easy and appeared compatible with people’s spontaneous intuitions. In our pre-teaching inclusion test, participants generally agreed that non-circle lines drawn on a sphere are not straight (average performance 94%), or that great circles are straight (average performance 84%). Accordingly, after the teaching phase, we found that all participants reached a good performance in these two conditions, independently of the number of lessons studied (average performance: non-circle lines 94%, great circles 97%). Performance was also good and was not affected by the number of lessons for non-planar non-straight lines (94%) and planar straight lines (90%) drawn on various surfaces—the equivalent of respectively non-circles and great circles drawn on a sphere.

In the two last test conditions, there was no effect of the teaching phase, and performance remained low in all groups, suggesting that the teaching we provided was not sufficient to solve these tasks. These two conditions require a high level of generalization with respect to the information presented in the lessons. For example, on some surfaces it is possible to find straight lines that are not planar; however this is not possible on the sphere, and consequently, this type of line was not exemplified in the lessons. Accordingly, participants generally failed to recognize this type of straight line when tested on various surfaces (average performance 33%), and the presentation of several lessons did not seem to help. Second, the number of lessons presented had little impact on participants’ reasoning about straight lines on arbitrary surfaces (average performance 67%). This last test condition included questions that required a high level of generalization (e.g., thinking about a cone to find an example of a straight line that intersects itself), as well as very intuitive questions that could be solved by thinking about the plane. Both these very easy and very hard trials presumably contributed to reducing the effects of the teaching condition in this test condition.

In summary, analyses of performance indicated that our participants benefited from the lessons presented and learned at least some aspects of the concept of generalized straight line. Our paradigm thus succeeded in creating conditions for studying the role of Eureka experiences in concept learning.

### Concept Learning Triggers Eureka Experiences

As a second conclusion, our experiment provides evidence that learning a new concept gives rise to Eureka experiences. At the end of the session, participants were asked whether they had experienced any Eureka moments: episodes where an idea came to them suddenly and unexpectedly, accompanied with a feeling of certainty. The description provided to our participants was inspired from a study assessing the sudden insights that arise as people are tasked to solve word puzzles (Jung-Beeman et al., 2004), and covered the phenomenological components classically associated with Eureka (or “Aha!”) experiences. After reading this description, a little over half of our participants reported experiencing Eurekas during the course of our experiment (61%).

Surprisingly, while the idea that students experience Eurekas in the science classroom appears to be widely shared amongst teachers (Brock, 2015), only one former study has attempted to survey Eureka experiences systematically in a population of (adult) mathematics students (Liljedahl, 2005). This study however suffers from severe methodological issues, which hampers any definitive conclusion from its results. First, reports of Eureka episodes were delayed in time until the end of a 13-week mathematics class, raising the risk of false memories. Second, and most importantly, students wrote little narratives describing their Eureka experiences in exchange of credits—and writing about a Eureka episode constituted an alternative to solving a math problem to earn these credits. Given that the population of students involved in the study (prospective teachers) reported high levels of math anxiety, this setting may have constituted a strong incentive for students to amplify or even fabricate false memories of Eureka experiences.

Importantly, in our study we incorporated several checks to ensure that Eureka reports were not purely fabricated by participants. First, we collected Eureka reports in the same session as the teaching and test phases, leaving limited time for false memories to emerge^7^. Second, participants were not rewarded for reporting Eurekas, and our instructions acknowledged that it is fully possible to learn a mathematical notion without experiencing any sudden epiphany. Third, and most importantly, we found evidence that participants’ reports of Eureka episodes were modulated by our experimental manipulation, in that the groups receiving more lessons were more likely to report Eurekas. This finding attests that Eureka reports did not simply reflect variations in personality traits, or in participants’ affinity with mathematics, but rather were causally related to the teaching we provided.

Fourth, Eureka experiences were related to performance in the post-teaching tests; and this relation was modulated across test conditions, in a pattern that held even when controlling for the number of lessons studied, years of education in mathematics, and participants’ confidence in their own understanding. This last finding allows us to exclude several deflationary explanations. For example, it is not the case that participants confabulated and reported imaginary Eureka episodes simply because they had studied many lessons and thought they ought to understand the notion taught very well, or because they had solved the post-teaching tests easily and felt confident about their own understanding.

In summary, our findings constitute the first robust empirical evidence that learning mathematical concepts can induce Eureka experiences—or at least, experiences that are remembered as Eurekas within the short time frame of a 60- to 90-minute experimental session. Note that so far, we only discussed the *occurrence* of a certain type of subjective experience, leaving aside the question of the reliability of the *content* of these experiences. For instance, while Eureka experiences leave learners with the impression that they have just made a great leap forward in understanding, these episodes may in fact be decoupled from actual learning, arising at random times when a learner is engaged with a difficult scientific concept. Furthermore, while learners’ experience is that of a sudden flash of understanding, we may find that they are able to access their own progresses at will and at all times, not only in rare Eureka episodes. These questions will be addressed in the next two sections.

### Eureka Experiences Signal Genuine Learning Progress

As described above, experiencing Eurekas was related to increased performance in specific subtests. In the previous section, we used this result to argue against deflationary explanations and establish a causal link between studying a concept and Eureka experiences. However, this finding also has stronger implications: it provides evidence that Eureka experiences do not simply arise when one merely engages with a novel concept, but rather signal genuine progress in learning.

According to the first-person description, learning occurs right when a Eureka is experienced: the learner was confused beforehand, and suddenly sees the light. It should be noted that, in our study, we did not attempt to assess whether Eureka experiences were accurate in their temporal aspect, and our findings are thus compatible with several alternative scenarios. For example, learning could actually be completed before the learner experiences a Eureka moment; more specifically, the knowledge acquired would remain implicit (but could still show in performance tests), until brought to the fore and made explicit in a Eureka moment (for studies testing this hypothesis in the domain of insightful problem solving, see e.g., Bowers et al., 1990; Durso et al., 1994; Ellis et al., 2011; Novick & Sherman, 2003; Smith & Kounios, 1996; a critical discussion of this line of studies can be found in Ash et al., 2009). Alternatively, it is also possible that Eurekas are felt before learning is actually completed: perhaps Eurekas are experienced during the initial steps of learning, the details of the episode being filled retrospectively once learning is complete. Again, this idea finds echoes in the literature, with theoretical views proposing that ideas gained by sudden insight are but a rough sketch or an initial hunch, further steps of evaluation and elaboration taking place after the initial discovery (e.g., Clement, 1989; Csikszentmihalyi & Sawyer, 1995; Hadamard, 1954; Ohlsson, 2009). All these alternatives are compatible with our findings, and deciphering the temporal relation between Eureka experiences and actual learning progress thus remains an interesting question for further research.

Our post-teaching tests used a variety of tasks to assess participants’ understanding of the concept of generalized straight line. Interestingly, we found that Eureka experiences predicted performance in one kind of generalization test: a test probing participants’ ability to identify counterintuitive straight lines of a type that had not been exemplified in the lessons (nonplanar straight lines on non-sphere surfaces). To succeed in this test condition, participants needed to overcome their naïve conception associating straightness with planarity, and instead apply the defining criteria for straight lines presented in the lessons. In contrast, however, Eureka experiences were not related to performance in a task testing a different kind of generalization: drawing inferences from the information taught to reason about the properties of straight lines on the sphere and on other surfaces. This pattern suggests that Eureka experiences were triggered as participants progressed in their understanding of the definitional properties of straight lines—identifying the core properties of the concept. Eureka experiences were not triggered however when participants reflected on the consequences of adopting this new definition—deriving the inferential role of the concept of straight line. This contrast may reflect a general property of learning-related Eureka experiences: Eureka experiences would be triggered specifically when learners engage in deep changes affecting the core definition of their concepts. Further research should be undertaken to test whether similar findings would obtain when people learn other mathematical concepts, or even concepts from other scientific domains.

### Dissociations between Eureka Experiences and Confidence

Our experiment also aimed at evaluating another aspect of the first-person Eureka experience: the idea that some concept learning processes (the processes susceptible to trigger Eureka experiences) may operate unconsciously, and remain inaccessible to reflective introspection. To that avail, besides asking participants to report on their Eureka experiences, we also asked them to reflect on their learning and evaluate their understanding of the concept of straight line.

Reflective judgments of confidence were dissociated from reports of Eureka experiences in our findings, at two levels. First, there was no correlation between Eureka reports and confidence ratings. Second, and most importantly, Eureka reports and confidence ratings were related to different patterns of performance in the post-teaching tests. In particular, the relation between Eureka experiences and straight lines identification described above held even after factoring out variations in participants’ confidence in their understanding. Learning abstract definitional properties of generalized straight lines thus involved processes that triggered Eureka experiences, yet did not inform participants’ reflective evaluation of their own understanding. Conversely, we also found that confidence ratings were uniquely associated with a change in performance, independently of Eureka reports. In detail, however, while confidence was correlated to performance in one of our analyses (analysis without covariates for number of lesson and education in mathematics), none of our test conditions showed a robust increase of performance in relation to confidence. It is thus hard to know whether the significant relation between confidence and performance reflects conceptual learning processes or processes implementing more superficial changes, such as changes in motivation or response strategies.

In summary, these findings establish the existence of dissociated processes respectively triggering Eureka experiences or informing people’s reflective judgments about their own understanding. However, only the processes associated with Eureka experiences were clearly implicated in conceptual learning.

The absence of a correlation between reports of Eureka experiences and confidence ratings may seem surprising, as this finding apparently contradicts the established relation between Eureka experiences and confidence (e.g., Danek & Wiley, 2017; Laukkonen et al., 2020). Our own instructions indeed emphasized confidence (feeling “certain”) as one key dimension of Eureka experiences. Participants’ explanations during debriefing proved particularly enlightening to understand this unexpected finding. In particular, we observed that, in a learning context, people may very well experience sudden flashes of insight involving a sensation of certainty, without feeling any more confident *about their understanding of the concept*. For example, one of our participants described a Eureka episode where she had suddenly realized that she did not understand straight lines. This episode was experienced as a Eureka moment because the participant felt suddenly certain of her own ignorance, but she certainly did not feel any more confident about her understanding of straight lines.

This example probably stands as an exception: the positive relation observed between Eureka reports and performance indicates that most Eureka episodes did contain information to advance people’s understanding of straight lines. Eureka episodes thus probably generally led participants to feel that they had progressed in their understanding of the concept of straight line, at least in the moment. However, perhaps this sensation of certainty was transient, and we failed to observe a correlation between Eureka experiences and confidence because we did not measure participants’ confidence at the very moment where Eureka experiences occurred. Suggestively, we found that confidence was highest when measured just after the teaching phase (linear mixed model on confidence, main effect of measurement time, *F*(2, 105.2) = 29.9, *p* < .001), i.e., when most Eureka experiences had just occurred (when asked to report when exactly they had experienced Eurekas, participants identified a total of 65 episodes, 44 of which were situated during the inclusion or teaching phase) and participants had not yet confronted their understanding to the generalization tests. Knowledge that has been gained by sudden insight may be particularly susceptible to interference from further testing: just like people solving problems by insights cannot describe how they found the solution (Bowden, 1997; Maier, 1931; Schooler & Melcher, 1995), in learning contexts Eureka experiences may convey knowledge without conscious access to any epistemic justification. Hence, while learners may be able to generalize the knowledge they gain by insight to new situations, doing so may lead them to realize that they cannot explain why these inferences are founded, and thus to lose confidence in their understanding.

In terms of mechanisms, our findings suggest that conceptual learning may involve an interplay between progressive learning processes operating outside the scope of consciousness, and consciousness acting as a discrete filter for access to learned information (for a model presenting consciousness as a discrete filter on perceived information, see Dehaene, 2014; Dehaene et al., 2003; for a similar idea applied to insightful problem solving, see Bowers et al., 1990; Ellis et al., 2011; Jung-Beeman et al., 2004; Zhong et al., 2008). Conceptual learning has been modelled computationally as a progressive accumulation process (e.g., Bonawitz et al., 2019; Gopnik & Wellman, 2012), where the learner would constantly evaluate how her conceptual representations fit with newly received information, and engage in conceptual change when a competing representation overcomes the current one. Eureka experiences could reflect key computational steps in these models: they could be triggered for example when a competing representation reaches a certain threshold (Eureka about a new understanding), or when the current representation drops to a floor level (Eureka about one’s ignorance).

Under this view, while learning may progress incrementally at the unconscious level, overt changes in concepts would necessarily involve the experience of a sudden leap of understanding. Interestingly, performance in our most difficult test of straight line identification (non-planar straight lines on non-sphere surfaces) appears coherent with this suggestion: participants did not succeed in this condition unless they had experienced a Eureka moment (see Figure 5). We suggest that the particular difficulty of concept learning may reside in this interplay between unconscious and conscious mechanisms. Hence, learning mechanisms may rarely reach the stage where progresses are made accessible to consciousness, such that students appear to stagnate for long periods of time. Moreover, after a Eureka experience has broadcasted a new leap in understanding, unless this insight is supported by external feedback it remains isolated, not grounded in epistemic justifications, and thus the progress achieved are particularly fragile.

## CONCLUSION

We developed a paradigm where participants were taught and tested on a novel mathematical concept, and analyzed participants’ reports of Eureka experiences in regard to their learning performance. More than half of our participants reported experiencing Eureka moments during the experimental session; and these episodes signaled genuine learning achievements, in the sense that participants who experienced Eurekas hold a more accurate and more generalizable representation of the concept taught than those who did not experience Eurekas. Moreover, the progresses associated with Eureka experiences failed to inform participants’ reflective judgments about their own learning. Our findings thus provide evidence that the Eureka experiences that arise while learning mathematical concepts are accurate, in two senses: they reflect the functioning of concept learning processes, and these concept learning processes appear to be inaccessible to reflective introspection.

Our study constitutes a first step in the investigation of learning-related Eureka experiences, and thus raises several questions for future research. For instance, are the Eureka experiences observed in the contexts of concept learning and problem solving qualitatively different, or do they reflect similar psychological processes? Furthermore, if all Eureka experiences turn out to indicate the successful termination of a search process, what is the nature of the search involved in conceptual learning, when all the necessary information has been provided explicitly by a teacher? Lastly, what kinds of learning achievements are related to Eureka experiences, and could a better understanding of the nature of these experiences and the conditions under which they arise help design more effective teaching methods for science and mathematics? We hope that our research will spark interest in these questions.

## ACKNOWLEDGEMENTS

We thank Valeria Giardino, Arnaud Viarouge, Andrew Shtulman, Emmanuel Sander, Jérôme Sackur, Claire Sergent, Franck Ramus, Olivier Mascaro, Laurianne Cabrera, Lola de Hevia, Judith Vergne, Timothé Bonhoure and T. R. Virgil for useful discussions.

## FUNDING INFORMATION

This study was funded by an ANR grant to VI (JCJC Geometries, ANR-17-CE28-0011-01), and a doctoral grant from Sorbonne Université to CB.

## Notes

^1^ In order to help participants identify the concept presented as a generalization of planar straight lines, in our experimental material we used the common term “straight line” rather than the technical term “geodesic”. Accordingly, in the description of our paradigm, we will refer to the concept under study as that of “straight line”.^2^ Participants of the 1-/7- and 3-/5-lesson conditions were recruited in two successive batches. Note that the average number of lessons was the same in these two batches (4 lessons), so differences between batches could not induce biases in our analyses, where number of lessons was encoded as a numerical variable. For more information about our recruitment and testing plan, see the Supplementary Online Material.^3^ For the sake of readability, here we only describe the tasks that are analyzed in this paper. A comprehensive list of the tasks presented to the participants can be found in the Supplementary Online Material.^4^ Afex’ function mixed is built on glmer from lme4. To assess the significance level of each fixed effect, mixed performs a likelihood test comparing the full glmer model to restricted models obtained by setting the parameters corresponding to the effect under study to 0. In all the statistics reported here, categorical variables were sum-coded, such that main effects and interactions were assessed while averaging over the levels of the categorical variables of non-interest. Consequently, the results reported are independent of the choice of a particular baseline level for categorical variables.^5^ We initially attempted to fit models with random slopes by test condition, with or without correlation terms, but the parameter estimation process failed to converge for these more complex models. We thus decided to revert to a simple intercept random effect structure in all our analyses. Discussions on how to set the random effect structure in mixed model analyses can be found in Barr et al. (2013), Bates et al. (2018), and Matuschek et al. (2017).^6^ Function emmeans uses the betas of the full-model glmer fit from afex’ mixed to compute estimated marginal means. Similarly, emtrends estimates the marginal slopes associated to numerical variables. Importantly, these marginal means and slopes can be estimated for each test condition, and the results are independent of the choice of a particular baseline level for test condition. All the statistics reported here average over the values of the variables of non-interest.^7^ At first view, it may have seemed tempting to instruct participants to keep an eye for Eureka episodes and report them on the spot, just as they arise. We opted against this solution, given evidence that asking people to watch their own mental processes can block performance specifically in so-called “insight problems”, i.e., problems that are typically solved by sudden insight (Schooler et al., 1993). We also feared that warning participants that they may experience Eurekas in the course of the experiment could lead them to expect these experiences to occur, thus increasing the rate of false positives.

## Supplementary Material


