# Supplementary material for: “Now I Get It!”: Eureka Experiences During the Acquisition of Mathematical Concepts
Source: Open Mind (Camb). 2024 Feb 1;8:17–41. doi: 10.1162/opmi_a_00116 (PMC10898616; doi:10.1162/opmi_a_00116)
Supplement: Supplementary file 1 [file opmi-08-17-s001.docx]

# Supplementary Material to “Now I get it !” : Eureka experiences during the acquisition of mathematical concepts

Charlotte Barot, Louise Chevalier, Lucie Martin, and Véronique Izard

This document has four parts. First, we present our general testing plan: how participants were assigned to the different experimental conditions, and which preliminary analyses were run as testing progressed. Second, we provide a full description of our paradigm, detailing all the tasks that were administered to our participants. We also explain the reasons that led us to discard some of these tasks from the analyses presented in the associated paper. Third, we present detailed results for the analyses presented in the associated paper; as well as for the same analyses applied to all the participants (including two groups not treated with a teaching phase, which were excluded from the main analyses; see ‘general testing plan’). Fourth, we present the full regression tables for all the analyses performed in the associated paper, for curious readers’ consideration.

# General testing plan

Testing was conducted in three batches. First, we administered the 1- and 7-lesson conditions in alternation, until we reached a sample size of 14 included participants in each group. The data of these two conditions was analyzed to check whether the teaching condition had an effect on test phase accuracy. We also inspected the data from the different tasks to check for any inconsistency, which led us to discard one task from analyses (see ‘detailed presentation of the tasks’). Crucially, at this first stage we did not perform analyses on participants’ Eureka reports in relation to performance or confidence judgments – the main analyses reported in our paper.

As a second step, we tested 28 participants (15 women, age 18-48 years, Mean = 25.6 years, exact age missing for two participants, 2-8 years of education in mathematics after 10th grade, Mean = 4.2 years) in two baseline conditions without a teaching phase. The participants of the first baseline group were presented with the one-page document with examples of great circles that served as an introduction to the lessons for the teaching groups, while the participants of the second baseline group proceeded directly from the inclusion phase to the test phase. Although we initially planned to include these two groups in the analyses, we eventually decided to exclude them, because we realized that their inclusion blurred the conclusions that could be derived from our experiment. First, participants of the baseline groups potentially evaluated their confidence based on different criteria: for example, they could feel very confident about the idea that small circles drawn on spheres correspond to straight lines, since they were never told that small circles are not straight. Similarly, while participants in the baseline groups reported Eureka experiences, the insights they gained in these episodes could have qualitatively different contents compared to the insights of the other groups. Lastly, since the baseline groups did not receive any lessons to study, it is unclear whether they engaged in learning at all, and thus their responses may be unrelated to the question at stake in our experiment - the role of Eureka experiences in concept learning.

In the last part of this document, we present the results of our analyses with the baseline groups included. These analyses yield the same results as the analyses presented in the associated paper.

Finally, in a third step, we tested participants in the 3- and 5-lesson conditions, with participants assigned to these two conditions in alternation.

This testing plan was adopted because we suspected that it may be difficult to recruit enough participants to complete all the groups – a suspicion that proved right, as we had to stop data collection after the 3- and 5-lesson testing batch, and could not test participants with 2, 4 or 6 lessons. Yet, we wanted to be sure to be able to run meaningful analyses on our data even if we had to stop data collection at some point. Thus, across the different batches the average number of lessons studied was kept constant (an average of 4 lessons for the 1- and 7-lesson batch, the 3- and 5-lesson batch, and the 2- and 6- or 4-lesson batches that could have followed), with the exception of the baseline batch. Consequently, and given that we analyzed the effect of our teaching intervention (the only aspect of the experiment to vary across batches) by means of a numerical variable for number of lessons, differences in performance between batches could not be captured by this variable, and thus would not be spuriously attributed to the manipulation of the teaching phase.

Because the average number of lessons is different in the baseline batch, the supplementary analyses presented in the third part of this document may present biases, unlike the main analyses reported in the associated paper. For this reason, as well as for the reasons explained above, the reader is advised to consider these results with caution.

|  | Baseline groups  (N=28) | 1-lesson group  (N=14) | 3-lesson group  (N=14) | 5-lesson group  (N=14) | 7-lesson group  (N=14) |
| --- | --- | --- | --- | --- | --- |
| Planar geometry test (accuracy) | 98% (85% to 100%) | 95% (85% to 100%) | 98% (90% to 100%) | 95% (85% to 100%) | 98% (85% to 100%) |
| Straight lines identification on the sphere, inclusion phase (accuracy) | 66% (50% to 83%) | 68% (58% to 83%) | 70% (50% to 83%) | 65% (42% to 75%) | 68% (50% to 83%) |
| Age (years) | 25.6 years  (18y. to  48y.) | 25.7 years  (18y. to  41y.) | 23.7 years  (20y. to  30y.) | 28.2 years  (19y. to  43y.) | 23.9 years  (20y. to  27y.) |
| Gender | 15 Female / 13 Male | 10 Female / 4 Male | 11 Female / 3 Male | 11 Female / 3 Male | 8 Female / 6 Male |
| Education in mathematics (number of years after 10^th^ grade) | 4.2 years  (2y. to 8y.) | 4.1 years  (2y. to 7y.) | 3.9 years  (2y. to 6y.) | 3.1 years  (2y. to 7y.) | 4.6 years  (0y. to 7y.) |

Table S0. Demographic data and average performance on inclusion tests by teaching condition (baseline, 1-, 3-, 5- or 7-lesson), for participants included in the final groups. Since the two baseline groups were analyzed together in the supplementary analyses of part III, they were pooled together in this table.

# Full description of the paradigm

Our paradigm comprised four phases: an *inclusion* phase, a *teaching* phase, a *test* phase, and a final *Eureka* phase where participants were asked about Eureka experiences. Here we present the comprehensive list of tasks administered to the participants in each of these phases, including some tasks that were not described and analyzed in the associated paper.

| **Inclusion phase** | **Teaching phase** | **Test phase** | **Eureka phase** |
| --- | --- | --- | --- |
| *Definition of straight lines (1)*  Planar geometry test Straight lines identification on  spheres | Introduction: great circles  Lessons about straight lines on the sphere | Confidence judgment (1) *Subjective efficiency of the teaching phase* Straight lines identification on spheres  *Definition of straight lines (2)*  Straight lines identification on various surfaces  Confidence judgment (2) Reasoning about straight lines on spheres and on other surfaces  Confidence judgement  (3)  *Definition of straight lines (3)*  *Retrospective ratings of understanding* | Eureka report |

Table S1. Comprehensive list of all the tasks administered to the participants in the four phases of the experiment. The tasks highlighted in italics are not described in the associated paper.

# Inclusion phase

*Definition of straight lines*. Participants were first asked to write a definition for the notion of straight line. Analyses of the definitions produced over the course of the experiment (three definitions for each participant) are not included in the associated paper. They will be reported in another paper analyzing people’s intuitive concept of straight lines.

*Planar geometry test*. This test was adapted from Izard et al. (2011; planar geometry condition, questions 1-20)^^[[1]](#footnote-1)^^. Participants were first introduced to a planar surface, extending indefinitely, on which points and straight lines could be drawn. Straight lines were described as lines that never turn, neither on the left nor on the right, and that continue straight ahead indefinitely. After this introduction, participants were asked a series of twenty illustrated questions about the properties of straight lines on this infinite plane. Questions were presented both in writing and orally through an audio recording, and participants ticked their answer (yes or no) on a response sheet. Participants were included if they made no more than 3 errors.

*Straight lines identification on the sphere*. In each trial, participants were presented with a photograph of a sphere (a table tennis ball) with a line drawn on it, and were asked to indicate whether the line was “straight” or not. Three types of trials were presented: non-circles (non-straight; e.g. wavy line, line looping and crossing itself to form an 8 figure), great circles (straight), and small circles (non-straight, but typically judged to be straight by most adults). Each category counted 4 trials, for a total of 12 trials presented in a randomized order. Participants responded by pressing the ‘O’ key for yes (in French: ‘oui’) or the ‘N’ key for no (‘non’). They were included if they made at least two mistakes on the small circle trials (i.e. they incorrectly judged small circles to be straight lines) or if they made at least two mistakes on the great circle trials (i.e. they incorrectly judged that great circles are not straight).

# Teaching phase

*Introduction to great circles*. Participants were given a one-page document defining the “great circles” of a sphere as circles that have the same radius as the sphere on which they are drawn. This introduction also provided illustrations of great circles drawn at various orientations. A translated version of this document is accessible on the Github repository of the project [(https://github.com/charlusb/Analyses_Eurekamaths)](https://github.com/charlusb/Analyses_Eurekamaths).

*Lessons*. Participants were given 1 to 7 lessons about straight lines in spherical geometry. The lessons used simple physical models to explain why great circles correspond to straight lines on the sphere, but smaller circles do not. These models were: rolling a toy car on a ball (the car can be rolled along a great circle, but not along a small circle; 2 lessons), applying scotch tape on a ball (the tape wrinkles if applied along a small circle, but remains smooth if applied along a great circle; 2 lessons), pinning a rubber band on a ball (the rubber band naturally aligns with a great circle; 2 lessons), and flight routes (flight routes seem curved when mapped on a planisphere, but prove to be the shortest route and a portion of a great circle when mapped on a globe; 1 lesson). A translated version of the lessons can be found on the Github repository of the project: [https://github.com/charlusb/Analyses_Eurekamaths.](https://github.com/charlusb/Analyses_Eurekamaths) For each teaching condition, different orders of presentation were created to ensure that each lesson appeared in each position, and that a given lesson was not always followed or preceded by the same lesson. To encourage participants to study the lessons in depth, we asked them to write a summary of each lesson just after reading it. We also asked them to rate whether they found the lesson convincing, on a scale graded from 0 to 10. These judgments of convincingness were collected twice: a first time after reading each lesson, and a second time for all lessons together (except for the group who received only one lesson to read, as this would have resulted in asking the exact same question twice in a row).

This and other tasks of the teaching phase (see Subjective efficiency of the teaching phase below) were included as an effort to develop a measure of the temporal dynamics of the learning process. Analyses of these tasks will be reported in a different paper, together with other experiments from that line of research.

# Test phase

*Confidence judgment*. Participants were asked to rate how much they felt they understood the notion of straight line, on a scale graduated from 0 to 10 (first rating of confidence).

*Subjective efficiency of the teaching phase*. Participants from the 3-, 5- and 7-lesson groups were given two questions to answer. First, they were asked whether they felt that the elements presented had helped them improve their understanding of straight lines (yes or no). Second, participants ranked the different lessons they had studied by mapping them on an oriented line, from the least convincing to the most convincing. This last task was not given to the 1-lesson group because they would have had only 1 lesson to rank. The first question about the subjective impact of the lessons on participants’ understanding was missing for this group by mistake.

At this point, the first experimenter left and was replaced by a second experimenter who was blind to the teaching condition assigned to the participants.

*Straight lines identification on spheres*. This task was identical to the spherical geometry inclusion task.

*Definition of straight lines.* Participants were asked to write a definition for the notion of straight line (second definition).

*Straight lines identification on various surfaces.* Participants were presented with lines drawn on four different surfaces: cone (8 trials), cylinder (6 trials), cube (8 trials) and torus (4 trials). Each trial displayed two photographs showing a front and a back view of a surface on which a line had been drawn. Participants were asked to judge whether the line presented was straight or not and indicated their answer by keypress.

The task crossed the two variables of straightness (straight, not straight) and planarity (planar, non-planar): 4 trials presented non-planar non-straight lines (corresponding to non-circle lines on the sphere), 3 trials presented planar straight lines (corresponding to great circles on the sphere), 10 trials presented planar nonstraight lines (corresponding to small circles on the sphere), and 9 trials presented non-planar straight lines (there are no corresponding examples on the sphere, but such lines can exist on other surfaces). Trials were presented in a random order.

*Confidence judgment.* Participants were asked to indicate how much they felt they understood the notion of straight line on a 0-10 graduated scale (second rating of confidence).

*Reasoning about straight lines on the sphere and on other surfaces.* This task consisted in a list of mathematical assertions, which participants judged to be true or false: eight assertions about the straight lines of the sphere, followed by eight assertions about straight lines on arbitrary surfaces. The assertions were presented in a fixed order, on paper. Participants were given written definitions for the terms ‘parallel’ and ‘perpendicular’, which appeared in some of the assertions. They answered by ticking one of four response options for each assertion: ‘yes - certain’, ‘yes - uncertain’, ‘no - uncertain’, and ‘no - certain’.

*Confidence judgment.* Participants indicated how much they felt they understood the notion of straight line on a 0-10 graduated scale (third rating of confidence). One participant from the 1-lesson group inadvertently failed to answer this third confidence question.

*Definition of straight lines.* Participants were asked to write a definition for the notion of straight line (third definition).

*Retrospective judgement of confidence.* Participants were asked to evaluate retrospectively their understanding of straight lines at three time points: before the teaching phase, at the end of the teaching phase and at the end of the test phase.

This task was originally included as another measure of participants’ reflective introspection about their own learning: we intended to measure how much people thought that they had progressed in their understanding of straight lines during the course of the experiment. However, inspection of the data of the first two groups (1 and 7 lessons, see ‘general testing plan’) revealed that participants often misunderstood this task. Indeed, if they had responded as intended, i.e. by indicating how much they thought they understood the notion of generalized straight line at several stages of the experiment *in the light of the understanding they had gained at the end of the experiment*, their ratings should progressively increase, or perhaps remain stable – but they cannot decrease. Quite the contrary, we found that the ratings produced by 19 out of 28 participants expressed a decrease in understanding at some point during the experiment. This suggests that many participants misinterpreted our question as referring to their *feeling of understanding as they experienced it at different stages of the experiment* – perhaps they thought that we wanted to study whether they could faithfully remember these feelings retrospectively. We thus chose to discard this measure from our analyses, yet the task was included for all the participants, for the sake of consistency between groups.

# Eureka phase

The final phase was administered by the experimenter who had been in charge of administering the teaching phase.

*Eureka report.* This final phase aimed at measuring whether people had experienced Eurekas during the course of the experimental session. Participants were first given a description of the sensations associated with Eureka experiences (adapted from Jung-Beeman et al, 2004^^[[2]](#footnote-2)^^): episodes where a new understanding arises suddenly and unexpectedly, and is associated with a feeling of certainty. Participants indicated whether they experienced such episodes at some point during the experiment (yes or no) – this answer was used as our measure of Eureka report. Next, participants were presented with vignettes illustrating the different phases of the experiment (one vignette for each lesson, pre- and post-teaching task, confidence question, and definition of straight lines), so that they could indicate when exactly in the experiment they had experienced Eurekas. A few participants from the 3- and 5-lesson groups were also asked to describe the insights that occurred to them in these occasions.

While testing the first two groups (1 and 7 lessons), we realized that the timing reported for Eureka episodes was sometimes quite unclear. For instance, some participants could select a particular lesson because they remembered having a sudden insight *about* this lesson, while they did not remember when exactly in the course of the study they had this insight. We thus decided against including the Eureka report by time point in our main analyses, but we nonetheless chose to include this task for subsequent testing batches, as it had proven very useful to obtain informal information about participants’ first-person experience in the study.

# Supplementary analyses

1. Detailed results of the main analyses

a. Effect of the number of lessons on test phase performance

The logistic mixed model analysis reported in the associated paper identified a significant interaction between number of lessons and test condition on participants’ test phase accuracy (χ²(8) = 36.2, *p* < .001).

Table S2 explores this significant interaction by examining the linear effect of number of lessons in each test condition.

# Linear effect of number of lessons on accuracy in each test condition

| Test Condition | Estimated Trend | Standard Error | 95%  Confidence  Interval,  Lower | 95%  Confidence  Interval,  Upper | *Z-*  ratio | Holm  corrected *p*-value |
| --- | --- | --- | --- | --- | --- | --- |
| Non-circle lines on spheres | 0.11 | 0.13 | -0.25 | 0.48 | 0.87 | 1.00 |
| Great circles on spheres | 0.15 | 0.19 | -0.37 | 0.67 | 0.79 | 1.00 |
| **Small circles on spheres** | **0.51** | **0.09** | **0.27** | **0.75** | **5.93** | **< .001** |
| Non-straight non-planar lines on various surfaces (eq. non-circle lines on spheres) | 0.29 | 0.15 | -0.12 | 0.70 | 1.98 | .28 |
| Straight planar lines on various surfaces (eq. great circles on spheres) | 0.03 | 0.12 | -0.31 | 0.37 | 0.24 | 1.00 |
| **Non-straight planar lines on various surfaces (eq. small circles on spheres)** | **0.16** | **0.05** | **0.01** | **0.31** | **3.03** | **.017** |
| Straight non-planar lines on various surfaces (no eq. on spheres) | 0.03 | 0.06 | -0.13 | 0.18 | 0.45 | 1.00 |
| **Reasoning on the sphere** | **0.18** | **0.06** | **0.02** | **0.35** | **3.14** | **.014** |
| Reasoning on various surfaces | 0.11 | 0.06 | -0.05 | 0.28 | 1.90 | .28 |

Table S2. Estimated linear trend for the effect of number of lessons on accuracy in each test condition. Model formula: Accuracy ~ TestCondition*NumberLessons + TestCondition*MathEducation + (1|Participant). The variable encoding number of lessons was centered on 0, and varied numerically between -3 (1 lesson) and +3 (7 lessons). Significant trends are highlighted in bold. Results are given in logit scale.

b. Relation between Eureka experiences and test phase performance

In the associated paper, we reported a significant interaction between a variable indicating whether participants had reported a Eureka experience and the variable for test condition on participants’ test phase accuracy (analysis without covariates: _χ²_(8) = 27.1, *p* < .001, analysis with covariates for number of lessons and education in mathematics, χ²(8) = 34.4, *p* < .001). Table S3 explores these significant interactions by examining the estimated contrast in accuracy between participants who did vs. did not report Eureka experiences in each test condition.

# Effect of Eureka report on accuracy in each test condition

| Test Condition | Estimated Contrast | Standard Error | 95%  Confidence  Interval,  Lower | 95%  Confidence  Interval,  Upper | *Z-*ratio | Holm  corrected *p*-value |
| --- | --- | --- | --- | --- | --- | --- |
| Non-circle lines on spheres | 0.20  *0.08* | 0.59  *0.64* | -1.45  *-1.69* | 1.84  *1.84* | 0.33  *0.12* | 1.00  *1.00* |
| Great circles on spheres | -1.18  *-1.75* | 1.12  *1.20* | -4.29  *-5.09* | 1.94  *1.58* | -1.05  *-1.46* | 1.00  *1.00* |
| **Small circles on spheres** | **0.96**  *0.39* | **0.35**  *0.38* | **0.00**  *-0.67* | **1.92**  *1.45* | **2.78**  *1.02* | **.043**  *1.00* |
| Non-straight non-planar lines on various surfaces (eq. non-circle lines on spheres) | -0.90  *-1.31* | 0.70 *0.74* | -2.84  *-3.38* | 1.03 *0.75* | -1.29  *-1.76* | 1.00 *.63* |
| Straight planar lines on various surfaces (eq. great circles on spheres) | -0.81  *-0.95* | 0.63 *0.67* | -2.56  *-2.81* | 0.94 *0.90* | -1.28  *-1.43* | 1.00 *1.00* |
| Non-straight planar lines on various surfaces (eq. small circles on spheres) | 0.25  *-0.04* | 0.26 *0.26* | -0.47  *-0.77* | 0.97 *0.69* | 0.97  *-0.15* | 1.00 *1.00* |
| **Straight non-planar lines on various surfaces (no eq. on spheres)** | **1.06** ***1.22*** | **0.29** ***0.30*** | **0.27** ***0.39*** | **1.86** ***2.05*** | **3.72** ***4.09*** | **.002**  ***< .001*** |
| Reasoning on the sphere | 0.36  *0.13* | 0.27  *0.28* | -0.40  *-0.65* | 1.12  *0.92* | 1.32  *0.47* | 1.00  *1.00* |
| Reasoning on various surfaces | 0.12  *-0.03* | 0.28  *0.29* | -0.66  *-0.83* | 0.90  *0.78* | 0.44  *-0.10* | 1.00  *1.00* |

Table S3. Estimated contrast in accuracy between participants who did vs. did not report a Eureka experience in each test condition. Plain: Model without covariates; formula: Accuracy ~ TestCondition*Eureka + (1|Participant). *Italics: Model with covariates for number of lessons and education in mathematics; formula: Accuracy ~*

*TestCondition*Eureka + TestCondition*NumberLessons + TestCondition*MathEducation + (1|Participant).* Significant contrasts are highlighted in bold. Results are given in logit scale.

c. Relation between Eureka experiences, confidence and test phase performance

The main analyses identified a significant interaction between Eureka report and test condition (analysis without covariates: _χ²_(8) = 26.1, *p* = .001, analysis with covariates for number of lessons and education in mathematics: _χ²_(8) = 34.2, *p* < .001), and between confidence and test condition (analysis without covariates: χ²(8) = 33.0, *p* < .001, analysis with covariates: χ²(8) = 25.9, *p* = .001) on participants’ test phase accuracy. Tables S4 and S5 explore these interactions.

# Effect of Eureka report on accuracy in each test condition (controlling for confidence)

| Test Condition | Estimated Contrast | Standard Error | 95%  Confidence  Interval,  Lower | 95%  Confidence  Interval,  Upper | *Z*-ratio | Holm  corrected *p*-value |
| --- | --- | --- | --- | --- | --- | --- |
| Non-circle lines on spheres | 0.08  *0.06* | 0.60  *0.64* | -1.59  *-1.71* | 1.75  *1.83* | 0.13  *0.09* | 1.00  *1.00* |
| Great circles on spheres | -1.13  *-2.53* | 1.26  *1.69* | -4.62  *-7.21* | 2.37  *2.16* | -0.89  *-1.50* | 1.00 *.94* |
| Small circles on spheres | 0.79  *0.32* | 0.36  *0.38* | -0.20  *-0.75* | 1.78  *1.38* | 2.22  *0.83* | .21  *1.00* |
| Non-straight non-planar lines on various surfaces (eq. non-circle lines on spheres) | -0.97  *-1.29* | 0.70 *0.75* | -2.92  *-3.38* | 0.98 *0.79* | -1.38  *-1.72* | 1.00 *.68* |
| Straight planar lines on various surfaces (eq. great circles on spheres) | -0.77  *-0.92* | 0.64 *0.68* | -2.54  *-2.80* | 1.00 *0.96* | -1.20  *-1.36* | 1.00 *1.00* |
| Non-straight planar lines on various surfaces (eq. small circles on spheres) | 0.25  *-0.02* | 0.27 *0.27* | -0.49  *-0.76* | 0.98 *0.72* | 0.93  *-0.07* | 1.00 *1.00* |
| **Straight non-planar lines on various surfaces (no eq. on spheres)** | **1.15** ***1.26*** | **0.30** ***0.30*** | **0.32** ***0.43*** | **1.97** ***2.10*** | **3.87** ***4.19*** | **.001**  ***< .001*** |
| Reasoning on the sphere | 0.27  *0.10* | 0.28  *0.28* | -0.51  *-0.69* | 1.06  *0.89* | 0.97  *0.36* | 1.00  *1.00* |
| Reasoning on various surfaces | 0.13  *-0.01* | 0.29  *0.29* | -0.68  *-0.82* | 0.93  *0.80* | 0.43  *-0.03* | 1.00  *1.00* |

Table S4. Estimated contrast in accuracy between participants who did vs. did not report a Eureka experience, in each test condition, controlling for confidence. Plain: Model without covariates; formula: Accuracy ~

TestCondition*Eureka + TestCondition*Confidence + (1|Participant). *Italics: Model with covariates for number of lessons and education in mathematics; formula: Accuracy ~ TestCondition*Eureka + TestCondition*Confidence +TestCondition*NumberLessons + TestCondition*MathEducation + (1|Participant).* Significant contrasts are highlighted in bold. Results are given in logit scale.

# Linear effect of confidence on accuracy in each test condition (controlling for Eureka report)

| Test Condition | Estimated Trend | Standard Error | 95%  Confidence  Interval,  Lower | 95%  Confidence  Interval,  Upper | *Z-*  ratio | Holm  corrected *p*-value |
| --- | --- | --- | --- | --- | --- | --- |
| Non-circle lines on spheres | 0.35  *0.32* | 0.22  *0.22* | -0.24  *-0.28* | 0.95  *0.92* | 1.64  *1.46* | .70  *1.00* |
| Great circles on spheres | -2.19  *-2.51* | 0.89  *1.18* | -4.66  *-5.78* | 0.28  *0.77* | -2.46  *-2.12* | .11  *.30* |
| Small circles on spheres | 0.34  *0.20* | 0.13  *0.14* | -0.03  *-0.18* | 0.70  *0.58* | 2.54  *1.45* | .099  *1.00* |
| Non-straight non-planar lines on various surfaces (eq. non-circle lines on spheres) | 0.18  *-0.01* | 0.21 *0.22* | -0.42  *-0.62* | 0.77 *0.60* | 0.82  *-0.06* | 1.00 *1.00* |
| Straight planar lines on various surfaces (eq. great circles on spheres) | -0.15  *-0.20* | 0.21 *0.22* | -0.74  *-0.81* | 0.44 *0.40* | -0.71  *-0.93* | 1.00 *1.00* |
| Non-straight planar lines on various surfaces (eq. small circles on spheres) | 0.00  *-0.06* | 0.10 *0.09* | -0.27  *-0.32* | 0.27 *0.21* | 0.04  *-0.59* | 1.00 *1.00* |
| Straight non-planar lines on various surfaces (no eq. on spheres) | -0.11  *-0.10* | 0.11 *0.10* | -0.40  *-0.39* | 0.18 *0.19* | -1.06  *-0.92* | 1.00 *1.00* |
| Reasoning on the sphere | 0.16  *0.09* | 0.10  *0.10* | -0.13  *-0.19* | 0.44  *0.37* | 1.51  *0.88* | .79  *1.00* |
| Reasoning on various surfaces | -0.01  *-0.06* | 0.11  *0.10* | -0.30  *-0.35* | 0.29  *0.23* | -0.07  *-0.60* | 1.00  *1.00* |

Table S5. Estimated linear trend for the effect of confidence on accuracy in each test condition, controlling for Eureka report. Plain: Model without covariates; formula: Accuracy ~ TestCondition*Eureka + TestCondition*Confidence + (1|Participant). *Italics: Model with covariates for number of lessons and education in mathematics; formula: Accuracy ~ TestCondition*Eureka + TestCondition*Confidence + TestCondition*NumberLessons +*

*TestCondition*MathEducation + (1|Participant).* The variable encoding confidence was centered, and varied numerically between -2.83 (confidence rating of 5/10) and +2.16 (confidence rating of 10/10). Significant trends are highlighted in bold. Results are given in logit scale.

2. Analyses including the participants who were not treated with a teaching phase

a. Effect of the number of lessons on test phase performance

# Logistic mixed model analysis of the effect of number of lessons on test phase accuracy

|  | df | χ² | *p* |
| --- | --- | --- | --- |
| **Test condition** | **8** | **1003.2** | **< .001** |
| **Number of lessons** | **1** | **23.7** | **< .001** |
| Education in Mathematics | 1 | 2.0 | .15 |
| **Test condition*Number of lessons** | **8** | **51.9** | **< .001** |
| **Test condition*Education in mathematics** | **8** | **19.0** | **.015** |

Table S6. Results of the logistic mixed model analysis of the effect of the number of lessons on test phase accuracy.

Shown here are likelihood ratio tests comparing the full model to restricted models lacking one of the predictors. Full model formula: Accuracy ~ TestCondition*NumberLessons + TestCondition*MathEducation + (1|Participant); Full model fit: LogLik = -2395.5, Random intercept (participant): variance = 0.27. Significant effects are highlighted in bold.

# Linear effect of number of lessons on accuracy in each test condition

| Test Condition | Estimated Trend | Standard Error | 95%  Confidence  Interval,  Lower | 95%  Confidence  Interval,  Upper | *Z*-  ratio | Holm  corrected *p*value |
| --- | --- | --- | --- | --- | --- | --- |
| Non-circle lines on spheres | 0.08 | 0.09 | -0.17 | 0.34 | 0.89 | 1.00 |
| **Great circles on spheres** | **0.40** | **0.14** | **0.01** | **0.78** | **2.82** | **.032** |
| **Small circles on spheres** | **0.40** | **0.06** | **0.24** | **0.56** | **6.96** | **< .001** |
| Non-straight non-planar lines on various surfaces (eq. non-circle lines on spheres) | 0.01 | 0.11 | -0.29 | 0.31 | 0.08 | 1.00 |
| **Straight planar lines on various surfaces (eq.**  **great circles on spheres)** | **0.23** | **0.08** | **0.00** | **0.45** | **2.83** | **.032** |
| **Non-straight planar lines on various surfaces (eq. small circles on spheres)** | **0.09** | **0.04** | **-0.00** | **0.19** | **2.67** | **.038** |
| Straight non-planar lines on various surfaces (no eq. on spheres) | 0.03 | 0.04 | -0.08 | 0.13 | 0.76 | 1.00 |
| **Reasoning on the sphere** | **0.12** | **0.04** | **0.01** | **0.22** | **3.03** | **.020** |
| Reasoning on various surfaces | 0.09 | 0.04 | -0.02 | 0.20 | 2.22 | .11 |

Table S7. Estimated linear trend for the effect of number of lessons on accuracy in each test condition. Model formula: Accuracy ~ TestCondition*NumberLessons + TestCondition*MathEducation + (1|Participant). The variable encoding number of lessons was centered, and varied numerically between -3 (1 lesson) and +3 (7 lessons). Significant trends are highlighted in bold. Results are given in logit scale.

b. Effect of the number of lessons on Eureka experiences

# Logistic regression analysis of the effect of number of lessons on Eureka report

|  | χ² | df | *p* |
| --- | --- | --- | --- |
| **Number of lessons** | **7.7** | **1** | **.005** |
| Education in mathematics | 0.2 | 1 | .63 |

Table S8. Results of the logistic regression analyzing the effect of the number of lessons on Eureka reports. Shown here are likelihood ratio tests comparing the full model to restricted models lacking one of the predictors. Full model formula: Eureka ~ NumberLessons + MathEducation. Full model fit: LogLik = -53.6. Significant effects are highlighted in bold.

c. Relation between Eureka experiences and test phase performance

# Logistic mixed model analysis of the relation between Eureka report and test phase accuracy

|  | No covariate analysis | | | Covariates analysis | | |
| --- | --- | --- | --- | --- | --- | --- |
|  | df | χ² | *p* | df | χ² | *p* |
| **Test condition** | **8** | **1017.5** | **< .001** | **8** | **1010.6** | **< .001** |
| **Eureka report** | **1** | **4.0** | **.046** | 1 | 0.9 | .34 |
| **Number of lessons** |  |  |  | **1** | **19.9** | **< .001** |
| Education in mathematics |  |  |  | 1 | 2.2 | .14 |
| **Test condition*Eureka report** | **8** | **16.5** | **.036** | **8** | **16.1** | **.041** |
| **Test condition*Number of lessons** |  |  |  | **8** | **51.9** | **< .001** |
| **Test condition*Education in mathematics** |  |  |  | **8** | **18.8** | **.016** |

Table S9. Results of the mixed model analyses of the relation between Eureka report and test phase accuracy. Shown here are likelihood tests comparing the full model to restricted models lacking one of the predictors. Left: analysis without covariates. Full model formula: Accuracy ~ TestCondition*Eureka + (1|Participant); Full model fit:

Loglik = -2430.6, Random effect (participant): variance = 0.33. Right: analysis with covariates for years of education in mathematics and number of lessons. Full model formula: Accuracy ~ TestCondition*Eureka +

TestCondition*NumberLessons + TestCondition*MathEducation + (1|Participant); Full model fit: LogLik = -2386.6, Random intercept (participant): variance = 0.26. Significant effects are highlighted in bold.

# Effect of Eureka report on accuracy in each test condition

| Test Condition | Estimated contrast | Standard Error | 95%  Confidence  Interval,  Lower | 95%  Confidence  Interval,  Upper | *Z*-ratio | Holm  corrected *p*-value |
| --- | --- | --- | --- | --- | --- | --- |
| Non-circle lines on spheres | 0.32  *0.22* | 0.46  *0.48* | -0.97  *-1.11* | 1.60  *1.55* | 0.68  *0.46* | 1.00  *1.00* |
| **Great circles on spheres** | **1.57**  *1.18* | **0.54**  *0.55* | **0.06**  *-0.35* | **3.07**  *2.71* | **2.89**  *2.14* | **.031** *.26* |
| Small circles on spheres | 0.56  *0.10* | 0.26  *0.29* | -0.17  *-0.70* | 1.29  *0.89* | 2.14  *0.34* | .23  *1.00* |
| Non-straight non-planar lines on various surfaces (eq. non-circle lines on spheres) | -0.76  *-0.74* | 0.61 *0.63* | -2.45  *-2.49* | 0.94 *1.00* | -1.24  *-1.18* | 1.00 *1.00* |
| Straight planar lines on various surfaces (eq. great circles on spheres) | 0.23  *-0.14* | 0.37 *0.38* | -0.79  *-1.20* | 1.25 *0.93* | 0.61  *-0.36* | 1.00 *1.00* |
| Non-straight planar lines on various surfaces (eq. small circles on spheres) | 0.16 *0.02* | 0.19 *0.19* | -0.37  *-0.51* | 0.69 *0.55* | 0.84 *0.09* | 1.00 *1.00* |
| **Straight non-planar lines on various surfaces (no eq. on spheres)** | **0.61** ***0.64*** | **0.21** ***0.21*** | **0.03** ***0.06*** | **1.19** ***1.22*** | **2.93** ***3.04*** | **.030**  ***.021*** |
| Reasoning on the sphere | 0.26  *0.13* | 0.21  *0.21* | -0.31  *-0.44* | 0.83  *0.70* | 1.28  *0.63* | 1.00  *1.00* |
| Reasoning on various surfaces | 0.17  *0.06* | 0.21  *0.21* | -0.41  *-0.53* | 0.76  *0.64* | 0.83  *0.27* | 1.00  *1.00* |

Table S10. Estimated contrast in accuracy between participants who did vs. did not report a Eureka experience, in each test condition. Plain: Model without covariates; formula: Accuracy ~ TestCondition*Eureka + (1|Participant). *Italics: Model with covariates for number of lessons and education in mathematics; formula: Accuracy ~*

*TestCondition*Eureka + TestCondition*NumberLessons + TestCondition*MathEducation + (1|Participant).* Significant contrasts are highlighted in bold. Results are given in logit scale.

d. Relation between Eureka experiences and confidence

i. Correlation tests

# Correlation table for confidence ratings and Eureka reports

|  | Confidence 1 | Confidence 2 | Confidence 3 | Eureka |
| --- | --- | --- | --- | --- |
| Confidence 1 | X | **ρ(84) = .67**  ***p* < .001** | **ρ(83) = .64**  ***p* < .001** | ρ(84) = .20 *p* = .17 |
| Confidence 2 | **ρ(82) = .64**  ***p* < .001** | X | **ρ(83) = .86**  ***p* < .001** | ρ(84) = .19 *p* = .17 |
| Confidence 3 | **ρ(81) = .65**  ***p* < .001** | **ρ(81) = .87**  ***p* < .001** | X | ρ(83) = .21 *p* = .17 |
| Eureka | ρ(82) = .12 *p* = .39 | ρ(82) = .15 *p* = .39 | ρ(81) = .21 *p* = .19 | X |

Table S11. Spearman’s ρ coefficients and *p*-values for pairwise correlation tests. Above diagonal: without covariates, below: with number of lessons and years of education in mathematics as covariates. Significant correlations are

highlighted in bold. All *p*-values were corrected for multiple comparisons using Holm’s method (applied separately

for the analyses with and without covariates). Note that the third rating of confidence was missing for one participant in the 1-lesson group, hence the difference in degrees of freedom. Confidence 1: measured just after participants completed the teaching phase; Confidence 2: measured after the various surfaces straight lines identification task; Confidence 3: measured after the reasoning task.

ii. Relation between Eureka experiences, confidence and test phase performance

# Logistic mixed model analysis of the relation between Eureka report, confidence and test phase accuracy

|  | No covariate analysis | | | Covariates analysis | | |
| --- | --- | --- | --- | --- | --- | --- |
|  | df | χ² | *p* | df | χ² | *p* |
| **Test condition** | **8** | **1025.6** | **< .001** | **8** | **1020.0** | **< .001** |
| **Eureka report** | **1** | **4.4** | **.036** | 1 | 1.6 | .21 |
| Confidence | 1 | 0.6 | .44 | 1 | 3.1 | .078 |
| **Number of lessons** |  |  |  | **1** | **21.3** | **< .001** |
| Education in mathematics |  |  |  | 1 | 2.2 | .14 |
| **Test condition*Eureka report** | **8** | **19.2** | **.014** | **8** | **18.6** | **.017** |
| **Test condition*Confidence** | **8** | **17.6** | **.024** | 8 | 14.1 | .080 |
| **Test condition*Number of lessons** |  |  |  | **8** | **48.1** | **< .001** |
| **Test condition*Education in mathematics** |  |  |  | **8** | **18.9** | **.015** |

Table S12*.* Results of the mixed models analyses of the relation between Eureka report, confidence and test phase accuracy. Shown here are likelihood ratio tests comparing the full model to restricted models. Left: analysis without covariates. Full model formula: Accuracy ~ TestCondition*Eureka + TestCondition*Confidence + (1|Participant);

Full model fit: LogLik = -2421.7, Random intercept (participant): variance = 0.33. Right: analysis with covariates for years of education in mathematics and number of lessons. Full model formula: Accuracy ~ TestCondition*Eureka +

TestCondition*Confidence + TestCondition*NumberLessons + TestCondition*MathEducation + (1|Participant); Full model fit: LogLik = -2378.6, Random intercept (participant): variance = 0.25*.* Significant effects are highlighted in bold.

# Effect of Eureka report on accuracy in each test condition (controlling for confidence)

| Test Condition | Estimated Contrast | Standard Error | 95%  Confidence  Interval,  Lower | 95%  Confidence  Interval,  Upper | *Z-*ratio | Holm  corrected *p*-value |
| --- | --- | --- | --- | --- | --- | --- |
| Non-circle lines on spheres | 0.26  *0.20* | 0.47  *0.48* | -1.04  *-1.13* | 1.55  *1.53* | 0.55  *0.42* | 1.00  *1.00* |
| **Great circles on spheres** | **1.82**  *1.50* | **0.56**  *0.58* | **0.27**  *-0.12* | **3.38**  *3.12* | **3.25**  *2.58* | **.011**  *.080* |
| Small circles on spheres | 0.48  *0.09* | 0.27  *0.29* | -0.26  *-0.70* | 1.22  *0.89* | 1.80  *0.33* | .51  *1.00* |
| Non-straight non-planar lines on various surfaces (eq. non-circle lines on spheres) | -0.77  *-0.74* | 0.62 *0.63* | -2.48  *-2.48* | 0.94 *1.01* | -1.25  *-1.17* | 1.00 *1.00* |
| Straight planar lines on various surfaces (eq. great circles on spheres) | 0.26  *-0.09* | 0.37 *0.39* | -0.78  *-1.16* | 1.29 *0.99* | 0.70  *-0.22* | 1.00 *1.00* |
| Non-straight planar lines on various surfaces (eq. small circles on spheres) | 0.15 *0.03* | 0.20 *0.19* | -0.39  *-0.51* | 0.69 *0.56* | 0.78 *0.13* | 1.00 *1.00* |
| **Straight non-planar lines on various surfaces (no eq. on spheres)** | **0.67** ***0.68*** | **0.21** ***0.21*** | **0.08** ***0.09*** | **1.26** ***1.27*** | **3.13** ***3.22*** | **.014**  ***.012*** |
| Reasoning on the sphere | 0.27  *0.16* | 0.21  *0.21* | -0.31  *-0.42* | 0.85  *0.73* | 1.31  *0.75* | 1.00  *1.00* |
| Reasoning on various surfaces | 0.20  *0.08* | 0.21  *0.21* | -0.40  *-0.50* | 0.79  *0.67* | 0.92  *0.40* | 1.00  *1.00* |

Table S13. Estimated contrast in accuracy between participants who did vs. did not report a Eureka experience, in each test condition, controlling for confidence. Plain: Model without covariates; formula: Accuracy ~

TestCondition*Eureka + TestCondition*Confidence + (1|Participant). *Italics: Model with covariates for number of lessons and education in mathematics; formula: Accuracy ~ TestCondition*Eureka + TestCondition*Confidence + TestCondition*NumberLessons+ TestCondition*MathEducation + (1|Participant).* Significant contrasts are highlighted in bold. Results are given in logit scale.

# Linear effect of confidence on accuracy in each test condition (controlling for Eureka report)

| Test Condition | Estimated Trend | Standard Error | 95%  Confidence  Interval,  Lower | 95%  Confidence  Interval,  Upper | *Z-*  ratio | Holm  corrected *p*-value |
| --- | --- | --- | --- | --- | --- | --- |
| Non-circle lines on spheres | 0.16  *0.14* | 0.13  *0.13* | -0.20  *-0.23* | 0.52  *0.51* | 1.23  *1.05* | 1.00  *1.00* |
| **Great circles on spheres** | -0.51  ***-0.56*** | 0.19  ***0.18*** | -1.04  ***-1.07*** | 0.02  ***-0.06*** | -2.65  ***-3.07*** | .073  ***.019*** |
| Small circles on spheres | 0.13  *0.01* | 0.08  *0.08* | -0.08  *-0.22* | 0.35  *0.24* | 1.70  *0.10* | .72  *1.00* |
| Non-straight non-planar lines on various surfaces (eq. non-circle lines on spheres) | 0.04 *0.00* | 0.16 *0.17* | -0.41  *-0.48* | 0.50 *0.48* | 0.25 *0.01* | 1.00 *1.00* |
| Straight planar lines on various surfaces (eq. great circles on spheres) | -0.07  *-0.12* | 0.12 *0.12* | -0.39  *-0.44* | 0.26 *0.20* | -0.57  *-1.04* | 1.00 *1.00* |
| Non-straight planar lines on various surfaces (eq. small circles on spheres) | 0.01  *-0.02* | 0.06 *0.06* | -0.15  *-0.17* | 0.17 *0.13* | 0.21  *-0.37* | 1.00 *1.00* |
| Straight non-planar lines on various surfaces (no eq. on spheres) | -0.08  *-0.09* | 0.06 *0.06* | -0.25  *-0.25* | 0.09 *0.08* | -1.30  *-1.43* | 1.00 *1.00* |
| Reasoning on the sphere | -0.02  *-0.06* | 0.06  *0.06* | -0.19  *-0.23* | 0.15  *0.10* | -0.27  *-1.05* | 1.00  *1.00* |
| Reasoning on various surfaces | -0.04  *-0.07* | 0.06  *0.06* | -0.21  *-0.24* | 0.14  *0.10* | -0.58  *-1.14* | 1.00  *1.00* |

Table S14. Estimated linear trend for the effect of confidence on accuracy, in each test condition, controlling for

Eureka report. Plain: Model without covariates; formula: Accuracy ~ TestCondition*Eureka +

TestCondition*Confidence + (1|Participant). *Italics: Model with covariates for number of lessons and education in mathematics; formula: Accuracy ~ TestCondition*Eureka + TestCondition*Confidence +*

*TestCondition*NumberLessons + TestCondition*MathEducation+ (1|Participant).* The variable encoding confidence was centered, and varied numerically between -2.83 (confidence rating of 5/10) and +2.16 (confidence rating of 10/10). Significant trends are highlighted in bold. Results are given in logit scale.

**IV. Regression tables**

1. Effect of the number of lessons on test phase performance

# Logistic mixed model analysis of the effect of number of lessons on test phase accuracy

|  | Beta | Std Error |
| --- | --- | --- |
| Intercept (average of all variable levels) | 1.48 | 0.12 |
| NonCircles | 1.42 | 0.27 |
| GreatCircles | 2.34 | 0.40 |
| SmallCircles | -1.06 | 0.16 |
| NonStraightNonPlanar | 1.72 | 0.34 |
| NonStraightPlanar | -1.22 | 0.11 |
| StraightNonPlanar | -2.24 | 0.12 |
| StraightPlanar | 0.85 | 0.24 |
| ReasoningOtherSurfaces | -0.68 | 0.12 |
| NumberLessons | 0.18 | 0.05 |
| MathEducation | 0.10 | 0.06 |
| NumberLessons:NonCircles | -0.06 | 0.12 |
| NumberLessons:GreatCircles | -0.03 | 0.17 |
| NumberLessons:SmallCircles | 0.33 | 0.08 |
| NumberLessons:NonStraightNonPlanar | 0.12 | 0.13 |
| NumberLessons:NonStraightPlanar | -0.01 | 0.05 |
| NumberLessons:StraightNonPlanar | -0.15 | 0.05 |
| NumberLessons:StraightPlanar | -0.15 | 0.11 |
| NumberLessons:ReasoningOtherSurfaces | -0.06 | 0.05 |
| MathEducation:NonCircles | 0.02 | 0.14 |
| MathEducation:GreatCircles | -0.20 | 0.20 |
| MathEducation:SmallCircles | 0.04 | 0.09 |
| MathEducation:NonStraightNonPlanar | 0.30 | 0.17 |
| MathEducation:NonStraightPlanar | -0.13 | 0.06 |
| MathEducation:StraightNonPlanar | 0.00 | 0.06 |
| MathEducation:StraightPlanar | -0.04 | 0.13 |
| MathEducation:ReasoningOtherSurfaces | 0.00 | 0.06 |

Table S15. Regression table for the logistic mixed model analyzing the effect of number of lessons on accuracy; formula: Accuracy ~ TestCondition*NumberLessons + TestCondition*MathEducation + (1|Participant). The groups

of participants who did not receive lessons to study were not included in this analysis. Test condition is encoded as a sum-coded 9-level categorical variable (the average of all conditions serves as reference). Number of lessons is encoded as a numerical variable centered on 0, ranging from -3 (1 lesson) to +3 (7 lessons). Education in mathematics is encoded as a numerical variable centered on 0, ranging from -3.93 (no mathematics after 10^th^ grade) to +3.07 (7 years of mathematics after 10^th^ grade).

2. Effect of the number of lessons on Eureka experiences

# Logistic regression analysis of the effect of number of lessons on Eureka report

|  | Beta | Std Error |
| --- | --- | --- |
| Intercept | 0.51 | 0.30 |
| NumberLessons | 0.36 | 0.14 |
| MathEducation | -0.13 | 0.16 |

Table S16. Regression table for the logistic regression analyzing the effect of number of lessons on Eureka report; formula: Eureka ~ NumberLessons + MathEducation. The groups of participants who did not receive lessons to study were not included in this analysis. Number of lessons is encoded as a numerical variable centered on 0, ranging from

-3 (1 lesson) to +3 (7 lessons). Education in mathematics is encoded as a numerical variable centered on 0, ranging from -3.93 (no mathematics after 10^th^ grade) to +3.07 (7 years of mathematics after 10^th^ grade).

3. Relation between Eureka experiences and test phase performance

# Logistic mixed model analysis of the relation between Eureka report and test phase accuracy

|  | No covariate model | | Covariates model | |
| --- | --- | --- | --- | --- |
|  | Beta | Std Error | Beta | Std Error |
| Intercept (average of all variable levels) | 1.46 | 0.13 | *1.55* | *0.13* |
| NonCircles | 1.41 | 0.26 | *1.34* | *0.28* |
| GreatCircles | 2.60 | 0.50 | *2.72* | *0.56* |
| SmallCircles | -1.22 | 0.16 | *-1.17* | *0.18* |
| NonStraightNonPlanar | 1.61 | 0.31 | *1.93* | *0.40* |
| NonStraightPlanar | -1.23 | 0.12 | *-1.29* | *0.13* |
| StraightNonPlanar | -2.38 | 0.13 | *-2.49* | *0.14* |
| StraightPlanar | 1.04 | 0.28 | *0.94* | *0.29* |
| ReasoningOtherSurfaces | -0.68 | 0.13 | *-0.75* | *0.14* |
| EurekaYes | 0.00 | 0.13 | *-0.13* | *0.13* |
| NumberLessons |  |  | *0.19* | *0.05* |
| MathEducation |  |  | *0.09* | *0.06* |
| EurekaYes:NonCircles | 0.09 | 0.26 | *0.16* | *0.29* |
| EurekaYes:GreatCircles | -0.59 | 0.50 | *-0.75* | *0.53* |
| EurekaYes:SmallCircles | 0.48 | 0.16 | *0.32* | *0.18* |
| EurekaYes:NonStraightNonPlanar | -0.46 | 0.31 | *-0.53* | *0.33* |
| EurekaYes:NonStraightPlanar | 0.12 | 0.12 | *0.11* | *0.13* |
| EurekaYes:StraightNonPlanar | 0.53 | 0.13 | *0.74* | *0.14* |
| EurekaYes:StraightPlanar | -0.41 | 0.28 | *-0.35* | *0.30* |
| EurekaYes:ReasoningOtherSurfaces | 0.06 | 0.13 | *0.11* | *0.14* |
| NumberLessons:NonCircles |  |  | *-0.09* | *0.13* |
| NumberLessons:GreatCircles |  |  | *0.09* | *0.18* |
| NumberLessons:SmallCircles |  |  | *0.29* | *0.08* |
| NumberLessons:NonStraightNonPlanar |  |  | *0.19* | *0.14* |
| NumberLessons:NonStraightPlanar |  |  | *-0.02* | *0.05* |
| NumberLessons:StraightNonPlanar |  |  | *-0.26* | *0.06* |
| NumberLessons:StraightPlanar |  |  | *-0.10* | *0.12* |
| NumberLessons:ReasoningOtherSurfaces |  |  | *-0.08* | *0.06* |
| MathEducation:NonCircles |  |  | *0.03* | *0.14* |
| MathEducation:GreatCircles |  |  | *-0.25* | *0.21* |
| MathEducation:SmallCircles |  |  | *0.06* | *0.09* |
| MathEducation:NonStraightNonPlanar |  |  | *0.27* | *0.17* |
| MathEducation:NonStraightPlanar |  |  | *-0.13* | *0.06* |
| MathEducation:StraightNonPlanar |  |  | *0.05* | *0.07* |
| MathEducation:StraightPlanar |  |  | *-0.06* | *0.13* |
| MathEducation:ReasoningOtherSurfaces |  |  | *0.00* | *0.07* |

Table S17. Regression table for the logistic mixed models analyzing the effect of Eureka report on test phase accuracy. The groups of participants who did not receive lessons to study were not included in these analyses. Left, plain: no covariate model; formula: Accuracy ~ TestCondition*Eureka + (1|Participant). Test condition and Eureka report are encoded as sum-coded 9- and 2-level categorical variables (the average of all levels serves as reference).

*Right, italics: model with covariates; formula: Accuracy ~ TestCondition*NumberLessons +*

*TestCondition*MathEducation + TestCondition*Eureka + (1|Participant). Again, test condition and Eureka report are encoded as sum-coded 9- and 2-level categorical variables (the average of all levels serves as reference). Number*

*of lessons is encoded as a numerical variable centered on 0, ranging from -3 (1 lesson) to +3 (7 lessons). Education in mathematics is encoded as a numerical variable centered on 0, ranging from -3.93 (no mathematics after 10^th^ grade) to +3.07 (7 years of mathematics after 10^th^ grade).*

4. Relation between Eureka experiences and confidence

# Logistic mixed model analysis of the relation between Eureka report, confidence and test phase accuracy

|  | No covariate model | | Covariates model | |
| --- | --- | --- | --- | --- |
|  | Beta | Std Error | Beta | Std Error |
| Intercept (average of all variable levels) | 1.77 | 0.22 | *1.94* | *0.28* |
| NonCircles | 1.20 | 0.34 | *1.03* | *0.39* |
| GreatCircles | 4.94 | 1.54 | *5.69* | *2.09* |
| SmallCircles | -1.50 | 0.24 | *-1.54* | *0.31* |
| NonStraightNonPlanar | 1.32 | 0.36 | *1.53* | *0.47* |
| NonStraightPlanar | -1.55 | 0.22 | *-1.68* | *0.28* |
| StraightNonPlanar | -2.71 | 0.23 | *-2.89* | *0.29* |
| StraightPlanar | 0.74 | 0.34 | *0.59* | *0.39* |
| ReasoningOtherSurfaces | -0.99 | 0.22 | *-1.14* | *0.29* |
| EurekaYes | -0.01 | 0.13 | *-0.17* | *0.15* |
| Confidence | -0.16 | 0.13 | *-0.26* | *0.15* |
| NumberLessons |  |  | *0.22* | *0.06* |
| MathEducation |  |  | *0.12* | *0.07* |
| EurekaYes:NonCircles | 0.05 | 0.27 | *0.20* | *0.30* |
| EurekaYes:GreatCircles | -0.55 | 0.56 | *-1.10* | *0.75* |
| EurekaYes:SmallCircles | 0.41 | 0.16 | *0.33* | *0.19* |
| EurekaYes:NonStraightNonPlanar | -0.47 | 0.31 | *-0.48* | *0.34* |
| EurekaYes:NonStraightPlanar | 0.13 | 0.13 | *0.16* | *0.15* |
| EurekaYes:StraightNonPlanar | 0.59 | 0.14 | *0.80* | *0.16* |
| EurekaYes:StraightPlanar | -0.37 | 0.28 | *-0.29* | *0.31* |
| EurekaYes:ReasoningOtherSurfaces | 0.07 | 0.14 | *0.16* | *0.16* |
| Confidence:NonCircles | 0.51 | 0.21 | *0.58* | *0.23* |
| Confidence:GreatCircles | -2.03 | 0.79 | *-2.25* | *1.05* |
| Confidence:SmallCircles | 0.50 | 0.15 | *0.46* | *0.17* |
| Confidence:NonStraightNonPlanar | 0.34 | 0.21 | *0.25* | *0.23* |
| Confidence:NonStraightPlanar | 0.16 | 0.12 | *0.20* | *0.15* |
| Confidence:StraightNonPlanar | 0.05 | 0.13 | *0.16* | *0.16* |
| Confidence:StraightPlanar | 0.01 | 0.21 | *0.06* | *0.23* |
| Confidence:ReasoningOtherSurfaces | 0.15 | 0.13 | *0.20* | *0.16* |
| NumberLessons:NonCircles |  |  | *-0.15* | *0.13* |
| NumberLessons:GreatCircles |  |  | *0.29* | *0.30* |
| NumberLessons:SmallCircles |  |  | *0.24* | *0.09* |
| NumberLessons:NonStraightNonPlanar |  |  | *0.17* | *0.15* |
| NumberLessons:NonStraightPlanar |  |  | *-0.04* | *0.06* |
| NumberLessons:StraightNonPlanar |  |  | *-0.27* | *0.07* |
| NumberLessons:StraightPlanar |  |  | *-0.09* | *0.13* |
| NumberLessons:ReasoningOtherSurfaces |  |  | *-0.09* | *0.07* |
| MathEducation:NonCircles |  |  | *-0.00* | *0.15* |
| MathEducation:GreatCircles |  |  | *-0.00* | *0.37* |
| MathEducation:SmallCircles |  |  | *0.02* | *0.10* |
| MathEducation:NonStraightNonPlanar |  |  | *0.23* | *0.18* |
| MathEducation:NonStraightPlanar |  |  | *-0.16* | *0.07* |
| MathEducation:StraightNonPlanar |  |  | *0.02* | *0.08* |
| MathEducation:StraightPlanar |  |  | *-0.07* | *0.14* |
| MathEducation:ReasoningOtherSurfaces |  |  | *-0.03* | *0.08* |

Table S18. Regression table for the logistic mixed models analyzing the effects of Eureka report and confidence on test phase accuracy. The groups of participants who did not receive lessons to study were not included in this analysis. Left, plain: no covariate model; formula: Accuracy ~ TestCondition*Eureka + TestCondition*Confidence +

(1|Participant). Test condition and Eureka report are encoded as sum-coded 9- and 2-level categorical variables (the average of all levels serves as reference). Confidence is encoded as a numerical variable centered on 0, ranging from -2.83 (confidence rating of 5/10) and +2.16 (confidence rating of 10/10). *Right, italics: model with covariates; formula: Accuracy ~ TestCondition*Eureka + TestCondition*Confidence + TestCondition*NumberLessons +*

*TestCondition*MathEducation + (1|Participant). Again, test condition and Eureka report are encoded as sum-coded 9- and 2-level categorical variables (the average of all levels serves as reference), and confidence is encoded as a numerical variable centered on 0, ranging from -2.83 (confidence rating of 5/10) and +2.16 (confidence rating of*

*10/10). Number of lessons is encoded as a numerical variable centered on 0, ranging from -3 (1 lesson) to +3 (7 lessons). Education in mathematics is encoded as a numerical variable centered on 0, ranging from -3.93 (no mathematics after 10^th^ grade) to +3.07 (7 years of mathematics after 10^th^ grade).*

1. Izard, V., Pica, P., Spelke, E. S., and Dehaene, S. (2011). Flexible intuitions of Euclidean geometry in an

   Amazonian indigene group. *Proceedings of the National Academy of Sciences of the United States of America*, *108*(24), 9782–9787. https://doi.org/10.1073/pnas.1016686108 [↑](#footnote-ref-1)
2. Jung-Beeman, M., Bowden, E. M., Haberman, J., Frymiare, J. L., Arambel-Liu, S., Greenblatt, R., Reber, P. J., & Kounios, J. (2004). Neural Activity When People Solve Verbal Problems with Insight. *PLoS Biology, 2*(4), e97. https://doi.org/10.1371/journal.pbio.0020097 [↑](#footnote-ref-2)
